# Valorization of Olive Leaf Extract via Tailored Liposomal Carriers: Comparative Analysis of Physicochemical Features, Antioxidant Capacity, and Stability

**DOI:** 10.3390/ph18111639

**Published:** 2025-10-30

**Authors:** Jovan Baljak, Dragana Dekanski, Andrea Pirković, Ninoslav Mitić, Aleksandar Rašković, Nebojša Kladar, Aleksandra A. Jovanović

**Affiliations:** 1 Center for Forensic Medicine, Toxicology and Molecular Genetics, Clinical Center of Vojvodina, 21000 Novi Sad, Serbia; jovan.baljak@mf.uns.ac.rs; 2 Department of Pharmacy, Faculty of Medicine, University of Novi Sad, 21000 Novi Sad, Serbia; nebojsa.kladar@mf.uns.ac.rs; 3 Institute for the Application of Nuclear Energy INEP, University of Belgrade, 11080 Belgrade, Serbia; dragana.dekanski@inep.ac.rs (D.D.); andrea.pirkovic@inep.co.rs (A.P.); ninoslavm@inep.co.rs (N.M.); 4 Department of Pharmacology, Toxicology, and Clinical Pharmacology, Faculty of Medicine, University of Novi Sad, 21000 Novi Sad, Serbia; aleksandar.raskovic@mf.uns.ac.rs; 5 Center for Medical and Pharmaceutical Investigations and Quality Control, Faculty of Medicine, University of Novi Sad, 21000 Novi Sad, Serbia

**Keywords:** antioxidants, by-product, HPLC, liposomes, olive leaves, extract, phospholipids, polyphenols

## Abstract

**Background/Objectives**: Olive leaf (*Olea europaea* L.), a by-product of olive oil production, is rich in bioactive phenolics but limited in application due to poor solubility and stability. To improve their bioavailability, this study presents a comparative encapsulation strategy using three phospholipid-based liposomal systems (AL, PG90, and PH90) loaded with ethanolic olive leaf extract. **Methods**: Liposomes were characterized by physicochemical parameters, encapsulation efficiency (EE), antioxidant activity, morphology, release kinetics under simulated physiological conditions, and 60-day stability. To the best of our knowledge, this is the first direct comparison of AL, PG90, and PH90 matrices for olive leaf extract encapsulation. **Results**: HPLC and GC-MS confirmed successful encapsulation, with oleuropein showing the highest EE (up to 76.18%). PH90 favored retention of non-polar triterpenes, while AL and PG90 preferentially encapsulated polar flavonoid glycosides. FT-IR analysis verified extract integration into phospholipid bilayers. Antioxidant activity remained high in all loaded formulations, with negligible activity in empty liposomes. Extract-loaded systems exhibited reduced particle size, higher viscosity, and more negative electrophoretic mobility, enhancing colloidal stability. PG90 liposomes displayed the most stable mobility profile over 60 days. Transmission electron microscopy and nanoparticle tracking analysis revealed formulation-dependent vesicle morphology and concentration profiles. Release studies demonstrated significantly prolonged polyphenol diffusion from PG90 liposomes compared to the free extract. **Conclusions**: Phospholipid composition critically governs encapsulation selectivity, stability, and release behavior. Tailored liposomal systems offer a promising strategy to enhance the stability and delivery of olive leaf polyphenols, supporting their application in bioactive delivery platforms.

## 1. Introduction

The olive (*Olea europaea* L., Oleaceae) tree, the most well-known species of the Mediterranean area, has been cultivated for millennia and traditionally valued not only for its fruits and oil but also for its leaves [1]. Olive leaves have a long history of use in traditional medicine for managing various diseases, such as hypertension, diabetes, hyperlipidemia, and urinary tract infections [2,3]. Numerous phytochemical and pharmacological studies have increasingly substantiated these therapeutic properties, highlighting the olive leaf as a valuable source of bioactive secondary metabolites [4]. Among the compounds present in olive leaves, phenolic secoiridoids, particularly oleuropein, are the most abundant and biologically significant [5]. In addition to its pharmacological profile, the olive leaf has emerged as a valuable by-product of the olive oil industry. The shift towards sustainable valorization of agricultural waste has brought renewed attention to olive leaves as a natural source of functional ingredients [6,7]. Olive leaf extract is formulated in various ways for different applications, including dietary supplements, cosmetics, and pharmaceutical products, often involving encapsulation or incorporation into specific delivery systems to enhance its stability [8,9,10].

Oleuropein, primarily found in young leaves and unripe fruits [11], exhibits a wide spectrum of pharmacological activities, including potent antioxidant [12,13], anti-inflammatory [14], hypolipidemic [15], and antimicrobial effects [16]. Additionally, chemical profiling of olive leaf extracts revealed the presence of other phenolic compounds, such as hydroxytyrosol, verbascoside, ligstroside, and oleuropein aglycone, which contribute synergistically to the overall biological potential of the extract [5,17]. Pentacyclic triterpenes derived from olive leaves, such as oleanolic and maslinic acids, present as free acids or as aglycone precursors of saponins, have demonstrated a wide range of pharmacological activities, including hepatoprotective, anti-inflammatory, antioxidant, and anticancer effects [18,19]. Given the well-documented health-promoting properties of these two acids, it is anticipated that this specific fraction of olive leaf extract will attract growing scientific and commercial interest [20]. However, the therapeutic potential of oleuropein and other compounds from olive leaf extracts is significantly affected by their pharmacokinetic properties [21]. After oral administration, oleuropein is thought to remain stable in the stomach, pass through the small intestine without significant absorption or metabolism, and reach the colon, where it undergoes microbial biotransformation, primarily through enzymatic hydrolysis, ultimately yielding hydroxytyrosol, a metabolite with even greater antioxidant and anti-inflammatory activity [22,23]. Despite this, both oleuropein and hydroxytyrosol exhibit limited bioavailability due to their hydrophilicity and metabolic instability, resulting in rapid systemic elimination [24]. In addition, gut microbiota, particularly *Lactobacillus* and *Bifidobacterium* in the colon, further transform oleuropein via β-glucosidase and esterase activity. Hence, the practical application of olive leaf extract is still limited by issues of stability, solubility, and targeted delivery [25]. Although preclinical studies have demonstrated the potential health advantages of dietary polyphenols, the low stability and bioavailability, as well as the bitter taste of products containing polyphenol compounds, restrict their wider use [26,27].

These challenges have directed our research group towards formulation approaches such as encapsulation in liposomes, biocompatible nanocarriers capable of improving the stability, permeability, and controlled release of encapsulated compounds [28,29]. Lipid-based colloidal systems, especially liposomes, are among the most widely explored encapsulation platforms in biomedical applications [30]. These carriers, characterized by their spherical structure composed of lipid bilayers, offer both hydrophilic and lipophilic environments suitable for diverse bioactive compounds [31]. Owing to their tunable size, multilamellarity, biocompatibility, and capacity to enhance bioavailability, liposomes are increasingly recognized as versatile and effective drug delivery vehicles in modern medical research and therapy [32,33,34]. Liposomal delivery systems are particularly promising for plant-derived phenolics due to their ability to enhance absorption and bioavailability, and to protect sensitive compounds from premature degradation [35,36,37]. Apart from their protective role, the influence of liposomes on improving the performance of encapsulated compounds, decreasing toxicity and adverse effects, and enhancing controlled delivery and activity is particularly important [38].

Therefore, this research aimed to develop and characterize liposomal formulations encapsulating olive leaf extract to enhance the stability of its polyphenolic constituents, thereby potentially maximizing their delivery and biological potential. The liposomal carriers were designed to enhance the stability of phenolic compounds from olive leaf extract, compounds known for their strong antioxidant and anti-inflammatory properties. These properties are particularly relevant for preventing or managing oxidative stress-related disorders. The primary envisioned route of administration is oral, aiming to develop a nutraceutical or functional food supplement for systemic antioxidant support and combating oxidative stress-related diseases, such as metabolic syndrome, cardiovascular conditions, and chronic inflammation. This approach addresses the challenges of low stability and poor gastrointestinal absorption of phenolic compounds in their free form. Additionally, there is potential for future topical applications, particularly in dermatological or cosmeceutical formulations, where the antioxidant and anti-inflammatory properties may be beneficial for skin protection against oxidative damage and aging. The specific objective was to optimize the encapsulation of olive leaf extract in three liposomal formulations based on different phospholipid sources: AL liposomes utilizing phospholipids from Avanti Polar Lipids, PG90 liposomes employing granulated phospholipids from Lipoid, and PH90 liposomes incorporating hydrogenated phospholipids from Lipoid. While liposomal delivery systems have been previously explored for olive leaf extracts, existing studies typically rely on a single phospholipid type, the addition of cholesterol, or using a more complex liposome encapsulation technique, such as the thin film method [39,40,41,42], limiting insight into how lipid composition influences encapsulation behavior and functional performance. In contrast, our study presents a systematic comparative evaluation of three distinct phospholipid matrices, enabling the identification of how variations in lipid source and composition impact the efficiency of encapsulation, compound selectivity, antioxidant activity, morphological properties, and long-term stability. To our knowledge, this is the first study to directly compare these specific phospholipid systems for encapsulating olive leaf extract, providing valuable guidance for the rational design of plant-based nanocarriers. Furthermore, the selection of phospholipid types was based on their differences in acyl chain saturation, molecular packing behavior, and predicted interactions with polyphenolic compounds in olive leaf extract. These structural variations can significantly influence liposomal properties, such as membrane fluidity, potential of encapsulation, and stability. AL phospholipids are in the form of non-hydrogenated and well-defined solid flakes, predominantly composed of unsaturated fatty acids (e.g., linoleic and oleic acids), offering high purity and reproducibility. Their use provides a baseline system for comparison. PG90 is composed of natural, unsaturated phosphatidylcholine, which provides a more fluid bilayer. This can enhance interaction with hydrophilic and amphiphilic polyphenols, potentially improving encapsulation of more polar compounds. PH90, being hydrogenated (i.e., fully saturated), forms more rigid and stable bilayers, which are expected to improve retention of lipophilic compounds, such as triterpenes, and to enhance oxidative stability. These phospholipids were chosen to represent a spectrum of membrane characteristics, from fluid to rigid, and to assess how these properties influence the encapsulation, protection, stability, and antioxidant potential of olive leaf polyphenols with varying polarity. Following encapsulation, the study will assess and compare the encapsulated profiles of bioactive compounds from each liposomal type, identifying the most effective carrier system for the extract’s active constituents. This includes the assessment of physicochemical properties, such as Fourier Transform Infrared (FT-IR) spectra, density, surface tension, viscosity, liposome concentration, and the chemical composition of the formulations, as well as the encapsulation efficiency of compounds via qualitative and quantitative HPLC and GC-MS analyses. In addition, transmission electron microscopy (TEM) and release kinetic study in the Franz diffusion cell were performed. Furthermore, chemical profile, vesicle size, polydispersity index (PdI), electrophoretic mobility, and zeta potential were monitored over a 60-day storage period to evaluate the stability of liposomes.

## 2. Results and Discussion

### 2.1. Polyphenol Profile of Olive Extract and Extract-Loaded Liposomes and Encapsulation Efficiency

The quantitative polyphenol profile of pure olive leaf extract and extract-loaded liposomes was examined in the HPLC analysis (Table 1). The amounts of compounds in extract-loaded liposomes represent the sum of values from both encapsulated and non-encapsulated fractions of liposomes. In addition, values for encapsulating fractions of the mentioned extract into liposomes and the efficiency of the liposomal encapsulation process in entrapping the main bioactives from the olive leaf extract formulation were presented (Table 2). Representative HPLC-DAD chromatograms of the olive leaf extract and liposomal samples are provided as Supplementary Material (Figures S1 and S2). In addition, the structural formulas of oleuropein, oleacein, ligstroside, oleuropein aglycone, luteolin 7-O-glucoside, and apigenin-7-O-glucoside, detected and quantified in olive leaf extract, are presented in Figure S3.

Among the quantified phytochemicals, oleuropein was identified as the most abundant compound in both the olive leaf extract (11.18%) and extract-loaded liposomal formulations (8.71–9.82%). Furthermore, oleanolic acid (4.28%) and maslinic acid (0.58%) were present as major representatives of the pentacyclic triterpene class. In addition, notable concentrations were observed for luteolin 7-O-glucoside (0.98%) and apigenin-7-O-glucoside (0.52%), representing the dominant flavonoid constituents. These compounds constituted the majority of bioactive constituents in the extract, confirming their role as the principal components of the formulation. The obtained results are in line with previously published studies [22,43,44,45], as the levels of oleuropein and flavonoids identified in the ethanol extracts were within the ranges reported in the literature. The predominance of oleuropein aligns with literature data reporting it as the key bioactive compound in olive leaf extracts, valued for its strong antioxidant and anti-inflammatory properties [28,43,45]. Similarly, the presence of oleanolic and maslinic acids is consistent with earlier phytochemical profiles of *O. europaea* by-products, where these triterpenes were also highlighted for their pharmacological relevance, particularly in anti-tumoral and hepatoprotective applications [43,44,46]. Importantly, the retention of these compounds in the liposomal formulations suggests good encapsulation efficiency and stability, as previously reported for polyphenol-rich plant extracts encapsulated in carriers [46], further supporting the suitability of liposomal delivery systems for preserving and delivering complex phytochemical profiles.

The amount of all quantified compounds was lower in the extract-loaded liposomal formulations compared to the pure extract. Upon liposomal encapsulation, a general reduction in phenolic compound concentrations was observed in all formulations, which is a common phenomenon attributed to processing losses during hydration, purification, or sonication steps [39]. Nevertheless, oleuropein remained the dominant compound in all three liposome types: AL (98.28 µg/mg), PG90 (85.75 µg/mg), and PH90 (87.18 µg/mg). The corresponding EE for oleuropein was relatively high, ranging from 72.90% in AL liposomes to 76.18% in PG90 liposomes, indicating that this compound possesses amphiphilic properties favorable for its integration into the bilayer or aqueous core of liposomes [46].

The encapsulation behavior of the triterpenes, particularly oleanolic acid and maslinic acid, was strongly influenced by the type of phospholipids used in formulation. PH90 liposomes, which are based on hydrogenated saturated phospholipids, showed the highest retention for both triterpenes, encapsulating 42.79 µg/mg of oleanolic acid with an EE of 92.93%, and 3.06 µg/mg of maslinic acid with 49.26% efficiency. This suggests that the saturated bilayer in PH90 creates a more favorable hydrophobic environment for the retention of lipophilic molecules, an observation also highlighted in recent formulations utilizing rigid vesicle systems for lipophilic drug delivery [40,41].

On the other hand, AL and PG90 formulations demonstrated better compatibility with more polar compounds, such as flavonoid glycosides. Luteolin 7-O-glucoside was encapsulated with an efficiency of 88.25% in AL liposomes and 89.79% in PG90 liposomes, while apigenin-7-O-glucoside achieved 94.38% and 93.36% in the same formulations, respectively. These results suggest that flavonoid glycosides, owing to their polar character and glycosylated structure, preferentially localize within the aqueous interior or hydrophilic interface of the bilayer, consistent with previous observations made in yogurt-enriched liposomal matrices and nanoscale emulsions enriched with olive leaf polyphenols [42,47].

In contrast, low encapsulation efficiencies were observed for highly hydrophilic and low-molecular-weight compounds, such as hydroxytyrosol and chlorogenic acid. Hydroxytyrosol was encapsulated at 45.14% in AL liposomes and 52.92% in PH90 liposomes, whereas chlorogenic acid showed minimal retention, with only 0.02 µg/mg in AL liposomes (22.22%) and non-detectable levels in PG90 and PH90 liposomes. This inefficiency is likely due to their limited affinity for liposomal bilayers, which reduces the likelihood of stable entrapment and promotes diffusion during processing and storage [48,49].

Compared to the other types, the selection of phospholipid types had a significant impact on encapsulation outcomes. AL liposomes exhibited the highest efficiency for glycosylated flavonoids and moderately polar phenolics, suggesting optimal amphiphilic balance. PG90 liposomes exhibited moderate performance across a range of compounds, indicating a balanced system. In contrast, the PH90 formulation demonstrated exceptional affinity for lipophilic triterpenes but lower compatibility with polar molecules, confirming its preferential application for nonpolar compound delivery, as supported by structural studies on rigid vesicle encapsulation [44,45].

The data suggest that the interplay of compound polarity, phospholipid composition, and vesicle microenvironment influences the efficacy of olive leaf phenolics. High EE values obtained for flavonoid glycosides and oleuropein demonstrate the potential of liposomal systems to effectively stabilize and deliver olive phenolics, particularly when phospholipid type is appropriately matched to compound properties. These findings are consistent with recent encapsulation studies emphasizing targeted design of carrier systems based on compound-lipid compatibility [40,41,42,47].

After 60 days in the refrigerator, significant degradation was observed in oleuropein and hydroxytyrosol, particularly in PG90 and PH90 formulations, indicating their limited stability (Supplementary Materials, Tables S2 and S3). In contrast, flavonoids and triterpenes, particularly quercetin and oleanolic acid, showed increased concentrations, likely due to structural transformations or better retention in liposomal membranes. EE improved for these stable compounds, while chlorogenic acid was completely degraded. These findings highlight the protective role of liposomes for selected constituents during storage, with detailed quantitative results presented in Supplementary Material (Tables S2 and S3).

### 2.2. FT-IR Spectra of Olive Leaf Extract-Loaded Liposomes

**Figure 1:**
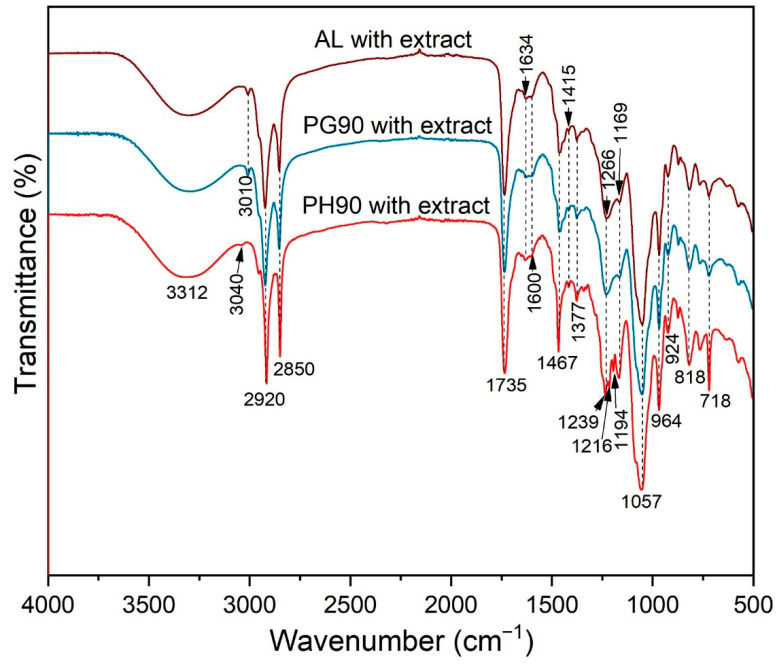
FT-IR spectra of olive leaf extract-loaded liposomes.

FT-IR spectroscopy was utilized to investigate the chemical composition of the developed extract-loaded liposomes. The corresponding spectra are shown in Figure 1. The FT-IR spectra of plain liposomes and olive leaf extract are presented in Figure S4.

The FT-IR spectra of plain liposomes confirmed the characteristic absorption bands of the phospholipid components (Figure S4A). Prominent peaks were observed around 2920 cm^−1^ and 2850 cm^−1^, corresponding to the asymmetric and symmetric stretching vibrations of CH_2_ groups in the lipid alkyl chains. The carbonyl (C=O) stretching vibration of ester groups appeared near 1735 cm^−1^, while phosphate-related vibrations were detected at ~1230 cm^−1^ (asymmetric PO_2_^−^ stretching) and ~1060 cm^−1^ (symmetric PO_2_^−^ stretching). These spectral features are consistent with the presence of intact phospholipid bilayers and indicate that the liposome preparation maintained the structural integrity of the lipid components. Comparison with the spectra of olive leaf extract-loaded liposomes further allows the identification of characteristic peaks associated with encapsulated bioactive compounds.

The FT-IR spectra of the olive leaf extract displayed characteristic peaks corresponding to its major bioactive compounds (Figure S4B). Broad absorption around 3400 cm^−1^ indicated the presence of hydroxyl (–OH) groups, typical of phenolics such as oleuropein and hydroxytyrosol. Peaks near 2920 cm^−1^ and 2850 cm^−1^ were attributed to CH_2_ stretching vibrations, while the strong band around 1735 cm^−1^ corresponded to carbonyl (C=O) stretching of ester and carboxylic groups. Additional bands at ~1600 cm^−1^ and ~1510 cm^−1^ were assigned to aromatic C=C stretching, confirming the presence of flavonoid and phenolic structures. Comparison of the FT-IR spectra of extract-loaded liposomes with those of plain liposomes and pure extract demonstrated the successful incorporation of olive leaf bioactives, as evidenced by the appearance of new peaks or shifts in characteristic bands, without disrupting the structural integrity of the phospholipid bilayer.

The FT-IR spectra confirmed successful encapsulation of olive leaf extract in phospholipid-based liposomes. Key peaks corresponding to the CH_2_ stretching vibrations (~2920 and ~2850 cm^−1^), carbonyl stretching (~1735 cm^−1^), and phosphate group vibrations (~1230 and ~1060 cm^−1^) were observed across all samples. Peaks at ~2920 cm^−1^ (asymmetric) and ~2850 cm^−1^ (symmetric) are presented due to CH_2_ stretching of lipid tails [50,51]. Non-hydrogenated liposomes (AL and PG90 samples) exhibit peaks that tend to be broader and may shift slightly due to the presence of unsaturated acyl chains, resulting in a more disordered bilayer. PH90 lipoosmes (hydrogenated) possess sharper peaks, slightly shifted to lower wavenumbers, which indicates more ordered, saturated chains. In AL and PG90 liposomes (non-hydrogenated), broader and slightly shifted CH_2_ peaks indicated increased bilayer fluidity, facilitating stronger interaction with olive leaf extract. This was further evidenced by shifts in the O–H and C=O regions, suggesting hydrogen bonding between the extract and the lipid headgroups. Conversely, PH90 liposomes (hydrogenated) exhibited more rigid, ordered bilayer features with minimal shifts, indicating limited interaction with encapsulated olive leaf extract due to tighter bilayer packing. Olive leaf extract contains polyphenols like oleuropein, which contribute to specific FT-IR peaks, including O–H stretching (3200–3500 cm^−1^), as the broad band due to hydrogen bonding in phenolic and hydroxyl groups, and C=O stretching (~1700–1740 cm^−1^), as carbonyl of lipid ester bonds and olive phenolic groups [52,53]. Encapsulation of olive extract’s bioactives may broaden or shift the O–H stretching band due to H-bonding interactions occurring with the lipid headgroups. Shift in the region of the C=O stretching suggests olive leaf extract-lipid interaction, likely via H-bonding or van der Waals interactions. Asymmetric PO_2_^−^ stretching at ~1230–1250 cm^−1^ and symmetric PO_2_^−^ stretching at ~1060 cm^−1^ are also visible in the FT-IR spectra of the developed liposomes, and shifts or intensity changes may indicate that olive leaf extract interacts with the polar head groups of phospholipids [50,54]. PH90 liposomes show a more ordered structure, leading to narrower C–H stretching bands and a possible blue shift (toward higher wavenumbers) in the phosphate region due to tighter packing, limiting olive bioactives’ penetration into the bilayer, therefore showing weaker shifts in O–H or C=O region compared to AL or PG90 liposomes, as well as less change in PO_2_^−^ bands [50,51]. On the other hand, the AL and PG90 liposomes bilayer allows more interaction with olive leaf extract, a stronger shift in C=O, and a broader O–H region, with possibly more change in the phosphate headgroup region. The presented data of the FT-IR spectra of the developed liposomes with olive leaf extract show the liposome composition via CH_2_ stretching bands, confirming lipid bilayers, and differences in these peaks between PH90 and AL or PG90 liposomes, supporting the saturation differences. Additionally, the data provide evidence of olive leaf extract encapsulation via the presence of O–H, C=O, and possibly aromatic C=C stretches (around ~1600 cm^−1^), and shifts or intensity changes in these peaks. The interaction of olive biactives with lipids is shown due to the shifts in phosphate and carbonyl regions, suggesting olive leaf extract-lipid head group interaction, which is more pronounced in AL and PG90 liposomes, due to higher fluidity and unsaturation. Regarding stability implications, PH90 liposomes may be more rigid and stable, but less efficient at encapsulating hydrophilic or amphiphilic olive leaf components (also proven in the HPLC analysis), while AL and PG90 liposomes may allow better encapsulation, but possibly less physical stability.

### 2.3. Antioxidant Capacity of Liposomes

**Figure 2:**
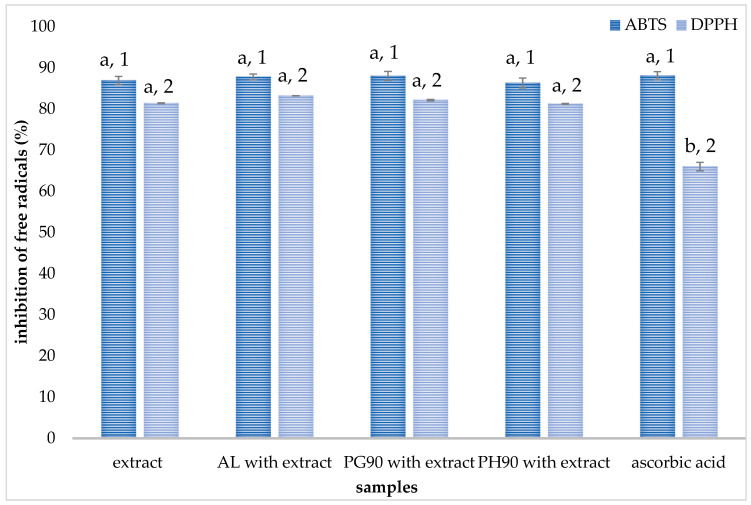
Antioxidant capacity of olive leaf extract and its liposomal formulations measured by ABTS and DPPH assays. Values with the same letter (per assay) and the same number (within each sample) show no statistically significant difference (*p* > 0.05; n = 3; one-way ANOVA with Duncan’s post hoc test). AL—liposomes prepared with phospholipids from Avanti; PG90—liposomes with granulated phospholipids (Lipoid); PH90—liposomes with hydrogenated phospholipids (Lipoid); ascorbic acid in a concentration of 400 µg/mL was used as the positive control.

The antioxidant capacity of olive leaf extract and plain liposomes, as well as extract-loaded liposomes, was investigated using ABTS and DPPH radical scavenging activity assays. The antioxidant activity of the liposomal formulations was assessed using ABTS and DPPH radical neutralization methods, which were selected for their simplicity, reproducibility, and widespread use in polyphenol analysis. Both methods are effective in detecting the free radical scavenging ability of hydrophilic and moderately lipophilic antioxidants and are particularly suitable for plant-based extracts formulated in various systems. The data from ABTS and DPPH tests are shown in Figure 2 (for olive leaf extract and extract-loaded liposomes) and in Figure S5 (for plain liposomes).

As can be seen from Figure 2, the anti-ABTS potential of the extract and liposomes with extract follows the trend: AL and PG90 > PH90 > extract, while statistical analysis showed the absence of significant differences. The highest antioxidant activity was provided by the extract-loaded PG90 liposomes (88.10%), followed by extract-loaded AL liposomes (87.76%). The ABTS radical scavenging potential of plain liposomes (arising from phospholipid manufacturer-added antioxidants) was 3.1–6.3% (Figure S5).

The DPPH radical neutralization capacity of both the olive leaf extract and liposomes with encapsulated extract followed a similar trend to that observed in the ABTS assay, with the order of activity being AL > PG90 > PH90 > extract, and no statistically significant differences were detected among the samples (Figure 2). The highest anti-DPPH potential was provided by the extract-loaded AL liposomes (83.20%), followed by extract-loaded PG90 liposomes (82.14%). All developed extract-loaded liposomes, as well as pure olive leaf extract, showed better radical scavenging capacity in the ABTS assay. As can be seen from Figure S5, plain liposomes showed very low antioxidant activity (2.15–5.62%).

The pure olive leaf extract exhibits high radical-scavenging activity in both ABTS and DPPH assays (ABTS: 86.92 ± 1.0%; DPPH: 81.44 ± 0.11%). These values reflect the well-known potent antioxidant capacity of olive polyphenols, particularly oleuropein and hydroxytyrosol, which are highly concentrated in olive leaves [55]. Polyphenols constitute the principal contributors to the antioxidant capacity of the olive leaf extract [56]. Several of these compounds have been individually assessed, and their antioxidant activity has been associated with the nature of their functional groups, their abundance, and the specific positioning of hydroxyl groups within their molecular structures, all of which confer antioxidant potential. Moreover, evidence indicates that phenolic constituents can exhibit synergistic interactions, enhancing overall antioxidant capacity when present in combination, as observed in olive leaf extract, compared to their isolated effects [56,57]. Literature data confirm similar in vitro antioxidant effects of olive leaf extract against diverse radical species, including peroxyl and superoxide radicals [56].

When the extract is incorporated into liposomal formulations, the ABTS results remain at high values (87–88%), and DPPH values are also sustained (81–83%). In contrast, the plain liposomes exhibit minimal radical scavenging activity, which clearly demonstrates that antioxidant activity stems from the extract and not from the liposomal carriers. The preservation of olive leaf extract’s antioxidant activity within liposomes aligns closely with literature findings, where González-Ortega et al. [48] and Ilgaz et al. [58] demonstrated that oleuropein-rich extracts encapsulated in soy phosphatidylcholine liposomes or niosomes exhibit strong antioxidant protection against lipid peroxidation, while Yuan et al. [59] encapsulated hydroxytyrosol (a major olive antioxidant) in liposomes and observed enhanced DPPH radical scavenging compared to free hydroxytyrosol, along with improved stability and controlled release. In our case, the similar antioxidant efficacy of the extract (diluted as in the case of liposomal formulation) and extract-loaded liposomes validates that the liposomal matrix preserves the radical-scavenging function of olive phenolics. The literature suggests two complementary mechanisms by which encapsulated olive phenolics confer antioxidant protection: direct radical scavenging and membrane stabilization. Polyphenols, such as oleuropein, donate hydrogen atoms to neutralize radicals, effectively halting chain propagation in oxidation, and can also increase lipid bilayer ordering, reducing the diffusion of radicals through the membrane and thereby offering steric and structural defense [48,55,58]. These dual mechanisms may explain why liposomal preparations with olive extract show maintained or slightly enhanced ABTS/DPPH scavenging compared to the free extract. The slight variations in the antioxidant capacity of various liposomal formulations were expected due to differences between phospholipid compositions, as well as their affinity to encapsulate different bioactive components depending on compound physicochemical properties (which is also shown through HPLC analysis, Section 3.1). Nevertheless, the absence of statistically significant differences between the radical scavenging potential of developed liposomes with encapsulated extract can be explained by the same amount of the extract (serving as the exclusive source of the observed antioxidant activity) in all liposomal formulations.

The preservation of antioxidant activity within liposomes underscores their suitability as delivery systems for olive leaf extract in formulations designed to combat oxidative stress, while similar results across AL, PG90, and PH90 liposomes suggest formulation flexibility, allowing selection based on encapsulation efficiency and stability without compromising antioxidant potency. Given that olive leaf extract carries additional health-promoting activities (e.g., anti-inflammatory, cardioprotective), liposomal encapsulation may support more effective delivery with improved bioavailability and stability. These findings are particularly relevant for functional food and nutraceutical applications, where maintaining antioxidant integrity during formulation and storage is critical. Furthermore, the consistent antioxidant performance across different phospholipid types suggests that lipid composition can be optimized based on other formulation goals, such as targeting specific release profiles or enhancing gastrointestinal stability, without compromising bioactivity. Future in vivo studies could further confirm whether this preserved in vitro antioxidant activity translates into enhanced biological efficacy and systemic protection against oxidative damage.

### 2.4. Stability of Liposomes During Storage

**Figure 3:**
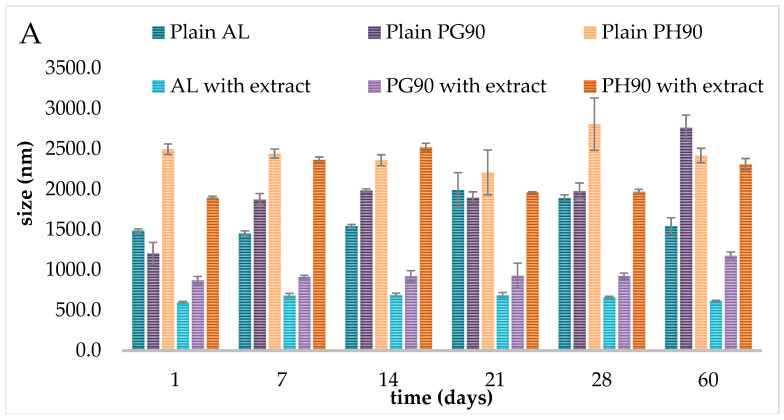

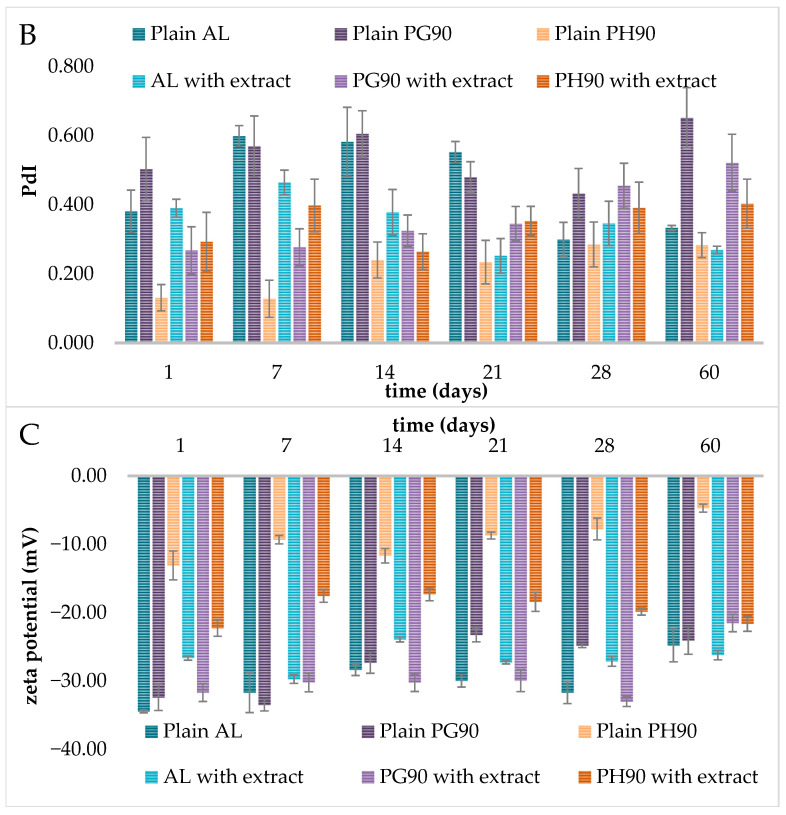
Time-dependent evaluation of physical stability parameters of plain and olive leaf extract-loaded liposomes over 60 days at 4 °C: (**A**) Z-average size, (**B**) polydispersity index (PdI), and (**C**) zeta potential. AL—liposomes prepared with phospholipids from Avanti; PG90—liposomes with granulated phospholipids (Lipoid); PH90—liposomes with hydrogenated phospholipids (Lipoid). Data represent mean ± SD of triplicate measurements.

The stability of liposomes during storage is a critical parameter for their suitability as drug delivery systems. In the present study, the stability of liposomes prepared with three different phospholipids, AL, PG90, and PH90, in both extract-loaded and empty forms, was examined over 60 days. The stability of plain and olive extract-loaded liposomes was monitored during storage in the refrigerator via measurements of their diameter, size distribution, electrophoretic mobility, and zeta potential using photon correlation spectroscopy. Thus, the results of liposome stability, in terms of size, size distribution, and surface charge during storage, are presented graphically in Figure 3. The data related to electrophoretic mobility are presented in Figure S6.

The diameter of all unloaded liposomes was significantly higher compared to extract-loaded parallels (Figure 3A). Immediately after preparation, the size of AL liposomes was 1487.7 ± 20.2 nm (plain) and 598.8 ± 10.8 nm (liposomes with extract), while the vesicle size of PG90 liposomes was 1204.0 ± 137.9 nm (plain) and 872.0 ± 46.7 nm (liposomes with extract). The differences between the size of PH90 plain and liposomes with extract were also significant, with values of 2494.3 ± 65.5 nm and 1896.7 ± 15.2 nm, respectively. The occurrence of larger liposomal particles in both cases (empty and olive extract-loaded liposomes, over 500 nm) was also expected since during the hydration stage in the proliposome technique, used in the development of these liposomes, the phospholipids swell and become hydrated, and it was also expected due to energy cost resulting in the creation of a closed lipid bilayer and very diverse multilamellar liposomes in terms of size and lamellarity [31,38]. The size of liposomes also plays a critical role in modulating their pharmacokinetic properties and half-life [31,33]. For example, smaller-sized lipid vesicles show a prolonged residence time in circulation compared to bigger particles that are eliminated more quickly from the bloodstream [60]. Larger liposomes up to 1000 nm (as olive leaf extract-loaded AL and PG90 liposomes) can be selectively absorbed by the reticuloendothelial system, and a significant amount was quantified in the spleen and liver. Furthermore, the observation that plain liposomes exhibited a larger particle size than extract-loaded liposomes can be attributed to several possible mechanisms, such as improved lipid packing, altered surface charge, effect on lipid phase properties, and enhanced hydration dynamics. This size discrepancy can be related to differences in membrane packing caused by the encapsulated extract, which may stabilize the bilayer structure and reduce vesicle fusion. Similar observations have been reported by Zhang et al. [61], who suggested that bioactive compounds can insert into the bilayer and restrict lipid movement, leading to more stable vesicle sizes. The bioactive constituents within the extract may integrate into the lipid bilayer, promoting tighter lipid arrangement. This enhanced molecular packing can lead to the formation of smaller, more stable vesicles by limiting membrane flexibility and curvature [62]. Certain molecules present in the extract might influence the surface charge (zeta potential) of the liposomes. A shift in surface charge can increase repulsive forces between liposomes during formation, thereby preventing aggregation and resulting in a smaller average size [63]. If the extract contains hydrophilic or amphiphilic components, it may change the hydration process of the lipid film during liposome preparation and therefore affect the usual high heterogeneity in size and lamellarity [38], i.e., it can provide smaller vesicles. Previous studies have shown that phenolic compounds can significantly impact the dynamics and organization of phospholipid assemblies. These effects stem from the interactions between lipids and various bioactive molecules, such as polyphenols, which can lead to modifications in liposome morphology, including changes in structural forms, particle dimensions, and trans-bilayer molecular transport mechanisms [64,65,66]. Research findings suggest that the incorporation of phenolic substances into liposomal carriers may reduce the availability of phospholipids for membrane formation, thereby producing lipid vesicles with reduced diameters. Specifically, polyphenolic compounds have been shown to enhance membrane fluidity, and this increased fluidity tends to support the formation of smaller liposomal structures [67]. Hence, these interactions between the extract and the lipid components during the formulation process may account for the reduced size observed in extract-loaded liposomes compared to the plain ones.

Additionally, a significant impact of the type of phospholipids on the size of the developed plain and olive extract-loaded liposomes was also noticed (Figure 3A). The phospholipid composition significantly influenced the initial vesicle size and its evolution during storage. Liposomes prepared with PH90, which contains a higher proportion of saturated phospholipids and is known for its high phase transition temperature, exhibited the largest size (2494.3 nm for the plain sample and 1896.7 nm for the extract-loaded sample). However, AL-based liposomes (likely rich in unsaturated phospholipids) displayed significantly smaller sizes (1487.7 nm for the plain sample and 598.8 nm for the extract-loaded sample). Extract-loaded liposomes prepared from PG90, which has an intermediate composition between AL and PH90, showed moderate initial sizes (~872.0 nm). In comparison, plain PG90-based liposomes have the lowest diameter among liposomal populations without extract (1204.0 nm). These findings align with previously reported studies, which indicate that the size of liposomal vesicles is influenced by the bilayer membrane composition as well as the properties and concentration of the encapsulated substances [68,69,70].

During the 60-day storage study, significant alterations in the liposome diameters can be noticed (Figure 3A). The phospholipid composition significantly influenced the alterations of liposome diameter during storage. Liposomes prepared with PH90, which contains a higher proportion of saturated phospholipids and is known for its high phase transition temperature, exhibited the largest sizes across all time points. On the 1st day, the extract-loaded PH90 liposomes had an average diameter of 1896.7 nm, which increased to 2309.0 nm by the 60th day. This increase suggests a propensity toward vesicle fusion or aggregation over time, consistent with previous studies highlighting the reduced fluidity of saturated bilayers and their limited capacity to accommodate encapsulated material without compromising structural integrity [71,72]. AL-based liposomes with extract (containing polyunsaturated fatty acids) displayed significantly smaller sizes (598.8 nm at the 1st day and 616.3 nm at the 60th day) and relatively stable size profiles, indicating better colloidal stability. This is in agreement with the findings of Leekumjorn et al. [73], who reported that unsaturated phospholipids maintain membrane fluidity and improve stability against aggregation. Liposomes prepared from PG90 showed moderate initial sizes (~872.0 nm for loaded liposomes) and a gradual increase over time (up to 1179.7 nm on the 60th day). These findings align with previous reports showing that phospholipid purity and saturation levels affect vesicle packing and long-term stability [71,72,74]. Empty liposomes prepared with AL showed moderate size fluctuations over the 60-day storage period. The vesicle size ranged from 1487.7 nm (day 1) to a maximum of 1990.0 nm (day 21), followed by a decrease to 1541.7 nm (day 60). This transient enlargement, followed by size reduction, suggests temporary aggregation or vesicle fusion, which later resolved, possibly due to sedimentation or rearrangement of lipid bilayers. However, there was no statistically significant difference between empty AL liposome sizes on the 1st and 60th days. These results align with prior studies showing that AL-based liposomes, rich in unsaturated phosphatidylcholines, maintain good stability under storage, due to flexible and fluid bilayers [71,72,73,75]. Empty PG90 liposomes exhibited more pronounced size increases and variability. Starting from 1204.0 nm on the 1st day, vesicle size rose sharply to 2761.7 nm by the 60th day (129% increase). The fluctuation suggests progressive aggregation or fusion of vesicles, consistent with literature indicating that the purity and partial saturation of PG90 phospholipids may predispose them to structural rearrangement over time [76]. The monitoring of particle size of lipid-based nanocarriers is important because the mentioned parameter plays a pivotal role in determining liposome stability, encapsulation capacity, drug release behavior, biodistribution, mucoadhesive properties, and cellular uptake [77]. The increase in vesicle size could also be attributed to oxidative degradation of unsaturated lipids or insufficient electrostatic or steric stabilization in the absence of encapsulated compounds [71]. Plain PH90 liposomes showed the largest initial vesicle sizes (2494.3 nm) and highly dynamic changes throughout storage. Size decreased to 2208.0 nm by the 21st day and then peaked at 2805.5 nm on the 28th day, followed by a reduction to 2416.7 nm on the 60th day. These oscillations may result from fusion–fission events, bilayer reorganization, or sedimentation of larger vesicle fractions [78]. Although PH90 consists primarily of hydrogenated phospholipids, which typically form rigid bilayers and improve shelf life, the large initial vesicle size and broad size variability suggest poor initial dispersion or susceptibility to aggregation. Viscosity plays a crucial role in the long-term storage stability of liposomal systems and is considered an important parameter for assessing their overall stability [79]. Hence, liposomes with higher viscosity exhibit reduced sedimentation rates, which helps maintain a consistent size distribution throughout storage, indicating enhanced formulation stability [80]. This relationship may explain the observed differences in stability between unloaded and olive leaf extract-loaded liposomes. Specifically, the increased viscosity observed in liposomes containing olive leaf extract (as presented in Section 2.7) likely hinders the aggregation of lipid vesicles, thereby minimizing significant changes in particle size over time.

In dynamic light scattering analysis, the focus is commonly on determining the size distribution of molecules, particles, or nanovesicles. This distribution reflects the relative abundance of vesicles within specific size ranges. PdI values describe the heterogeneity of particle sizes within liposomal formulations: PdI < 0.3 suggests monodisperse, uniform size distribution (desirable), while PdI values ranging from 0.3 to 0.7 represent moderate polydispersity [77,81]. Empty PH90 liposomes showed the lowest PdI, indicating the most homogenous vesicle population, whereas empty PG90 and AL liposomes had higher PdI values, suggesting more variability in vesicle sizes (Figure 3B). Significantly lower PdI of plain PH90 liposomes in comparison to plain AL and PG90 liposomes can be explained by a significantly higher Z-average size of the PH90 sample. Indeed, larger liposomal vesicles possessed lower values of PdI than reduced-sized liposomes [82, 83, 84]. Compared to the PdI value of AL unloaded liposomes (0.381 ± 0.062), AL liposomes with olive extract possessed a similar PdI, i.e., without a statistically significant difference (0.390 ± 0.026) (Figure 3B), indicating the presence of moderately heterogeneous dispersion (samples with PdI higher than 0.3) [85]. The PdI values of plain and extract-loaded PH90 liposomes were 0.131 ± 0.038 and 0.293 ± 0.085, respectively, indicating a high level of homogeneity (samples with PdI lower than 0.3). The PdI values of plain and extract-loaded PG90 liposomal systems were 0.503 ± 0.092 and 0.268 ± 0.068, respectively, indicating that the addition of olive leaf extract caused a decrease in the PdI value. As can be seen, the addition of olive extract did not trigger changes in the size distribution of the AL liposomal systems, which agrees with the literature data [64,77]. On the other hand, for the PH90 and PG90 liposomal populations, the influence of the extract was substantial and showed an opposite trend. Specifically, in the PH90 sample, the encapsulation of the extract increased the PdI value (resulting in a broader particle size distribution). In contrast, in the PG90 liposomes, the presence of the extract induced an increase in the homogeneity (narrower size distribution). The type of phospholipid and the presence of plant extract can significantly influence the PdI of liposomal formulations. PH90 formed the most uniform empty liposomes, but this uniformity was compromised with the addition of olive leaf extract. Conversely, AL and PG90, as more polydisperse in their plain forms, benefit from extract loading, potentially due to favorable bilayer interactions with extract constituents. The presence of the olive leaf extract may have destabilized the vesicle formation or caused variable vesicle sizes, possibly due to less compatibility between the extract and the lipid bilayer in PH90 liposomes, resulting in reduced homogeneity. Namely, interactions between the extract and the saturated lipid bilayer may destabilize uniformity, perhaps through partial bilayer disruption or altered packing [74,84]. On the other hand, the extract might have interacted more favorably with the lipid components of PG90, possibly stabilizing the vesicles or promoting uniform assembly. This contrast may stem from differences in lipid composition, bilayer packing, or phase behavior between PH90 and PG90 formulations.

Since liposomal stability during storage is a critical factor for their pharmaceutical and cosmetic applications, one of the key parameters used to monitor liposomal stability over time is the PdI, which reflects the size uniformity of vesicles. A low and stable PdI (<0.3) indicates good physical stability, whereas increasing PdI values may suggest vesicle aggregation, fusion, or leakage of contents. As can be seen in Figure 3B, over the 60-day storage period, most formulations in the current study displayed relatively stable PdI values, although some variability was noted depending on the lipid composition and the presence of the plant extract. Empty AL liposomes exhibited higher PdI values throughout the storage period (mean ~0.46), suggesting greater heterogeneity and lower physical stability. Empty PH90 liposomes maintained low PdI values (~0.23–0.28), indicating better colloidal stability, likely due to the saturated nature of the phospholipids and reduced bilayer fluidity, which limits vesicle fusion [31]. PG90 formulations, particularly the empty PG90 liposomes, showed that PdI increases over time (up to ~0.65), suggesting the onset of destabilization, possibly due to lipid oxidation or gradual aggregation. Incorporation of the plant extract appeared to influence liposomal stability [72,85,86]. Namely, for AL formulation, the presence of extract reduced PdI values and helped maintain better uniformity over 60 days, which suggests that extract constituents (e.g., polyphenols) may have stabilizing effects on the lipid bilayer. These compounds may act as antioxidants or bilayer reinforces [87,88]. However, PG90 liposomes with extract showed a higher increase in PdI over time, while the increase was lower in the extract-loaded PH90 liposomes, suggesting that extract-lipid interactions might destabilize the bilayer in lipid systems. The obtained results of PdI are in agreement with the changes in particle size. Hence, the loss of liposome stability was exacerbated by the increase in PdIs, as almost all diversified vesicular systems tend to aggregate during storage [89]. Over time, several physical processes can compromise liposome stability, including aggregation or fusion of vesicles, which leads to increased size and PdI, hydrolysis or oxidation of unsaturated phospholipids, which destabilizes bilayers, leakage of encapsulated compounds due to bilayer fluidity or structural defects, and reorganization of bilayer components (e.g., extract insertion or head group rearrangement), which alters surface charge and colloidal stability [71,76,90]. Such changes are often more pronounced at higher temperatures but may also occur at 425 °C over long durations. The 60-day storage stability of the liposomal formulations varied with lipid type and the presence of plant extract. Therefore, empty PH90 liposomes exhibited better stability in terms of PdI, while AL liposomes with extract showed improved stability, highlighting the protective effect of the extract. Moreover, empty PG90 and AL formulations were more prone to size heterogeneity and possible fusion over time. Maintaining storage temperature, utilizing antioxidants (such as polyphenols), and selecting suitable phospholipid types (e.g., hydrogenated or saturated ones like PH90) are crucial for the long-term stability of liposomes.

Due to their spherical and three-dimensional form, liposomes can provide higher levels of mobility and fluidity across the natural cell membrane [91,92]. According to Duffy et al.’s study [93], the movement of liposomes is a function of liposome composition, lipid vesicle diameter, and charge, which can explain the electrophoretic mobility differences between various developed liposomes in the present study. In addition, the mentioned parameter represents an important characteristic for liposome behavior and distribution in multiple formulations, simulated fluids, cell or animal models, as well as in the human body, to allow the release of bioactive compounds on the target sites, thus avoiding undesigned toxicity associated with the drug molecules [33,94,95,96]. Electrophoretic mobility correlates directly with zeta potential, a key predictor of colloidal stability. More negative values of electrophoretic mobility (e.g., <−1.5 µm/cm/Vs) usually indicate a higher surface charge, stronger repulsion, and better dispersion stability. Less negative or near-zero values suggest weaker electrostatic repulsion and a higher risk of aggregation. Therefore, it is important to investigate the electrophoretic mobility of developed liposomes.

Electrophoretic mobility is directly related to the surface charge of liposomes and serves as the basis for calculating zeta potential, a key indicator of colloidal stability. Monitoring electrophoretic mobility over time allows us to detect subtle changes in surface properties that may not be evident from size or zeta potential alone. In our study, electrophoretic mobility trends supported stability data and contributed to distinguishing differences between liposomal formulations during storage. AL with extract formulation consistently showed highly negative electrophoretic mobility values (from −1.87 µmcm/Vs to −2.33 µmcm/Vs, Figure S6), suggesting electrostatic stabilization similar to that of the empty AL formulation (from −1.94 µmcm/Vs to −2.70 µmcm/Vs). This may result from the natural presence of phosphate groups in soy-derived lecithin in AL, which contribute a negative surface charge [97] and incorporation of anionic compounds from the plant extract, further enhancing charge density on the vesicle surface [63]. Formulation with PG90 and extract also showed high negative electrophoretic mobility values (up to ~−2.58 µmcm/Vs, Figure S6), indicating a high surface charge and good electrostatic stabilization. This may be attributed to the inherent anionic nature of some extract components (e.g., flavonoids and phenolic acids) and improved bilayer organization or insertion of stabilizing molecules [84]. PH90 liposomes with extract showed less negative electrophoretic mobility (from ~−1.35 µmcm/Vs to ~−1.74 µmcm/Vs), suggesting moderate colloidal stability, which may result from the saturated nature of PH90 phospholipids, creating a rigid bilayer and may limit insertion of charged extract components, as well as possible surface shielding by extract molecules, leading to partial charge neutralization [98]. Empty PH90 liposomes had poor electrostatic stability and may require stabilizers for long-term use, while extract incorporation improves their stability, likely due to charge enhancement from phenolic compounds. Specifically, the lowest electrophoretic mobility values were seen in empty PH90 formulation during storage (as low as −0.37 µmcm/Vs), suggesting weak electrostatic repulsion, increasing the risk of aggregation and sedimentation, and the lack of negatively charged head groups or stabilizing agents in saturated phospholipids. This is consistent with observations that neutral or saturated lipids (e.g., hydrogenated soy phospholipids) alone provide minimal surface charge, compromising long-term colloidal stability [71]. Extract loading improved electrophoretic mobility (i.e., made values more negative) for PH90 liposomes. In the case of AL liposomes, the electrophoretic mobility decreased upon the extract encapsulation (from ~−2.70 µmcm/Vs to ~−2.09 µmcm/Vs). Schlieper et al. [99] have found that bound compounds decrease liposome electrophoretic mobility. The fraction of olive extract compounds not encapsulated within the liposomes may adhere to the exterior surface of the lipid bilayer, owing to established interactions between lipids and polyphenols [89,100]. This surface association can lead to a decrease in liposome electrophoretic mobility. Ruozi et al. [89] reported that the interactions among phospholipid head groups and compounds, as well as compounds’ absorption on the liposome surface, can restrict their electrophoretic mobility. The extract’s effect on the increase in electrophoretic mobility of PG90 liposomes suggests better compatibility or insertion of extract molecules into the rigid bilayer. Smaller and more fluid liposomal vesicles also show a higher electrophoretic mobility because of the increased deformability of the bilayer membrane [101]. Nevertheless, the more significant contribution of zeta potential to the liposomal electrophoretic mobility than that of the liposome size was demonstrated by Chen & Arriaga [102]. The movement of liposomes also depends on the surrounding temperature [94].

Over 60 days, empty PH90 liposomes demonstrated a marked decrease in electrophoretic mobility (absolute value), from approximately −1.02 µmcm/Vs to −0.37 µmcm/Vs, suggesting progressive destabilization due to surface charge reduction. This may be attributed to lipid oxidation or hydrolysis, which can disrupt bilayer integrity and cause the release or rearrangement of surface-exposed groups [71,103]. Similarly, empty PG90 liposomes showed a gradual decline in electrophoretic mobility, from −2.54 µmcm/Vs to −1.89 µmcm/Vs, indicating moderate surface charge attenuation. This trend may stem from the partial degradation of unsaturated phospholipids, leading to decreased electrostatic repulsion among vesicles [76]. Empty AL liposomes also showed a decrease in electrophoretic mobility but maintained highly negative electrophoretic mobility values (−2.70 µmcm/Vs to −1.94 µmcm/Vs), suggesting moderate storage stability. Despite the presence of unsaturated fatty acids in soy-derived lecithin, the surface charge appears to be more resistant to decay, possibly due to its natural phosphatidylcholine composition, which provides both electrostatic and steric stabilization [104]. The incorporation of olive leaf extract attenuated the electrophoretic mobility decay, particularly in PH90 and AL formulations. For instance, extract-loaded PH90 liposomes retained higher (more negative) electrophoretic mobility values (−1.74 µmcm/Vs to −1.69 µmcm/Vs) compared to their empty counterparts (−1.02 µmcm/Vs to −0.37 µmcm/Vs), suggesting that anionic compounds from the extract (e.g., phenolic acids or flavonoids) contributed to maintaining surface charge [63,105]. This phenomenon supports the hypothesis that bioactive plant constituents enhance colloidal stability via surface interactions. PG90 liposomes with olive extract exhibited consistent electrophoretic mobility values across the storage period (ranging from −2.48 µmcm/Vs to −1.69 µmcm/Vs), further affirming the stabilizing influence of extract constituents. AL liposomes with extract also demonstrated good charge retention (electrophoretic mobility values ranging from −2.09 µmcm/Vs to −2.06 µmcm/Vs), slightly more stable than empty AL liposomes (−2.70 µmcm/Vs to −1.94 µmcm/Vs). This suggests some degree of electrostatic reinforcement by extract components even in soy-derived systems. A drop in the electrophoretic mobility values during storage can be attributed to changes in liposome sizes, specifically their increase [94]. Additionally, in the case of plain liposomes, the population with the highest electrophoretic mobility also possessed the highest zeta potential value (Figure 3C and Table S6).

The incorporation of the plant extract into PH90 liposomal formulation resulted in increased (i.e., more negative) zeta potential values, indicating enhanced electrostatic stabilization. In the PH90 formulation, the zeta potential increased from −13.1 mV in the empty system to around −22.2 mV upon loading (Figure 3C). A different shift was observed in AL and PG90 formulations, with extract-loaded AL liposomes maintaining a zeta potential around −26.6 mV compared to −34.4 mV in the empty system. In comparison, there was no statistically significant difference between plain and extract-loaded PG90 liposomes (~−32 mV). The differences may be attributed to the presence of anionic phytochemicals in the extract, such as phenolic acids, which can insert into or adsorb onto the liposome surface, changing surface charge density [77,106]. Previous studies have shown that phenolic-rich extracts can significantly increase the negative surface charge of lipid vesicles, contributing to enhanced repulsive forces and colloidal stability [88,107]. The type of phospholipid also considerably influenced the zeta potential values of the developed liposomes. Specifically, empty AL and PG90 liposomes both exhibited highly negative zeta potential values (−34.4 mV and −32.4 mV, respectively), consistent with their natural content of phosphate head groups and unsaturated fatty acids, which contribute to surface charge and fluid bilayer structures [74]. Empty PH90-based liposomes exhibited significantly less negative zeta potential (−13.1 mV), likely due to their saturated phospholipid composition, which results in more rigid bilayers and less surface-exposed charge groups. These trends are in agreement with literature data showing that saturated lipids (such as hydrogenated phosphatidylcholine in PH90) result in tighter bilayer packing, reduced exposure of ionizable head groups, and lower zeta potential, while unsaturated lecithin in AL or PG90 liposomes confers a greater surface charge [71].

During 60 days of storage, zeta potential values declined in absolute values, particularly for the PH90 and AL formulations, suggesting a gradual reduction in colloidal stability. Empty PH90 liposomes showed the largest decline, dropping from ~−13.1 mV initially to as low as −4.7 mV, indicating possible vesicle aggregation, oxidation, or surface ion desorption over time. Plain AL and PG90 formulations also exhibited declines (from ~−34.4 mV to ~−24.8 mV, and from ~−32.4 mV to ~−24.1 mV, respectively), which could be attributed to oxidation of unsaturated phospholipids or rearrangement of surface components [71]. Interestingly, extract-loaded PG90 maintained relatively stable zeta potential values until the 28th day (~−33 mV), while the AL system with extract maintained same zeta potential value until the 60th day (~−26 mV) suggesting a protective effect of the extract, possibly through antioxidant activity stabilizing the bilayer or by maintaining surface charge via persistent binding of anionic compounds [108]. The zeta potential of PH90 liposomes with encapsulated extract varied in a very narrow range during storage (~−22.2 mV to ~−21.6 mV). These findings align with reports that liposomes with zeta potential values more negative than −30 mV are stable, while a decline below −15 mV can increase the likelihood of aggregation due to reduced electrostatic repulsion [77]. Despite relatively high values of the zeta potential of all AL and PG90 liposomes, according to the literature data, neutral or positively charged unilamellar or nanoliposomal particles are removed from the blood more slowly compared to giant and unmodified liposomal spheres [109]. Nevertheless, the presence of a strong electrostatic charge (positive or negative) can facilitate the interaction of opsonin-producing compounds, such as proteins, with liposomal particles and enhance their elimination from the bloodstream [110]. The charge characteristics of the liposomal membrane surface can significantly affect the potential interaction among liposomal vesicles and target cells. The alterations in the lipid composition of the liposomes can modify the surface charge, and the overall charge of liposomal particles may be adjusted by the implementation of charged compounds, resulting in a negative, positive, or neutral charge. Neutral liposomal vesicles (particles lacking surface charge) exhibit a greater tendency to aggregate, which restricts the physical stability of liposomes and minimizes their interactions with cells, leading to the extracellular release of bioactives [33]. On the other hand, charged liposomal particles, such as olive leaf extract-loaded liposomes, possess various advantages compared to neutral lipid vesicles. Due to a surface charge, electrostatic repulsion between liposomal particles prevents their aggregation and flocculation, facilitated by the presence of a zeta potential with either positive or negative values. Additionally, lipid particles with a high value of electrostatic surface charge exert a larger potential to provide interactions with target cells [33,111].

The observed differences in EE and stability among the AL, PG90, and PH90 liposomal formulations can be attributed to the structural and physicochemical properties of the phospholipids used. Similarly, the antioxidant activity in our study correlates with the retention of key polyphenols in the liposomal matrix, further highlighting the importance of tailoring lipid composition to the physicochemical properties of encapsulated bioactives. Phospholipid matrices differ in terms of saturation level, chain length, and packing behavior, which affect bilayer fluidity and permeability, key factors in the retention and protection of polyphenolic compounds. For example, the hydrogenated phospholipids in PH90 form more rigid and ordered bilayers, which likely contributed to the improved retention of lipophilic triterpenes. In contrast, unsaturated phospholipids (PG90 and AL samples) form more fluid bilayers, which can favor encapsulation of more polar flavonoid glycosides but may lead to greater permeability and compound leakage over time. These findings are consistent with the literature on phospholipid complex-based delivery systems, where lipid matrix rigidity and chain composition directly influence drug encapsulation, release kinetics, and bioavailability. Additionally, while doping with certain soft or transition metal ions has been reported in the literature to influence encapsulation efficiency in some systems, its effect is highly dependent on the type of bioactive compound, the metal, and the liposomal composition [112,113]. Investigating such strategies could be an interesting direction for future research to potentially enhance encapsulation performance.

### 2.5. Nanoparticle Tracking Analysis of Developed Liposomes

**Figure 4:**
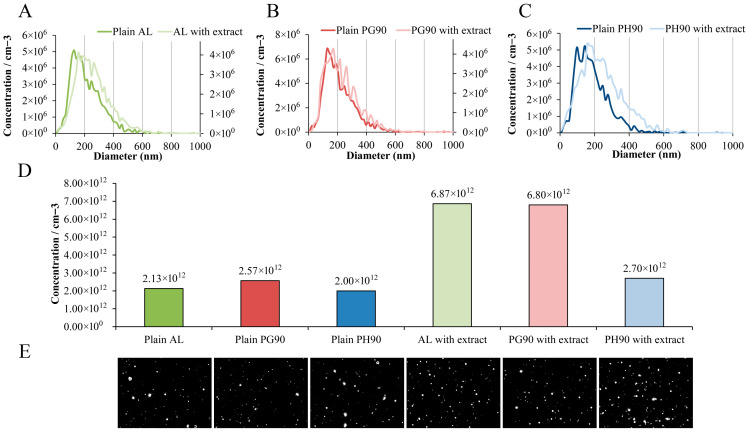
Nanoparticle tracking analysis (NTA) of liposomal formulations: (**A**–**C**) size distribution profiles, (**D**) particle concentration (particles/mL), and (**E**) representative video frame captures. Data represent mean values of three independent NTA measurements. AL—liposomes prepared with phospholipids from Avanti; PG90—liposomes with granulated phospholipids (Lipoid); PH90—liposomes with hydrogenated phospholipids (Lipoid).

NTA was employed to assess both the size distribution and particle concentration of blank and extract-loaded liposomal formulations. The resulting data are shown in Figure 4.

NTA of size distributions showed that developed liposome preparations exhibited similar size heterogeneity. Additionally, vesicle concentrations among the empty liposomes were relatively uniform, with plain AL, PG90, and PH90 showing 2.13 × 10^12^, 2.57 × 10^12^, and 2.00 × 10^12^ particles/mL, respectively. The extract-loaded AL and PG90 liposome preparations demonstrated the highest particle concentrations (6.87 × 10^12^ and 6.80 × 10^12^ particles/mL, respectively), while extract-loaded PH90 liposomes showed a lower concentration (2.70 × 10^12^ particles/mL), similar to the plain PH90 liposome preparation. All liposome formulations were prepared using the same concentrations of phospholipids and olive leaf extract. However, due to inherent differences in the physicochemical properties of the phospholipid types used (e.g., chain saturation, molecular packing), and resulting variations in vesicle size, the final liposome concentrations (number of particles/mL) differed between formulations. This data is important as it reflects the structural output of each lipid system, helping to evaluate how phospholipid composition affects vesicle formation and potential dose uniformity in delivery applications.

Broad size distributions are characteristic of conventional liposome preparations, particularly when using passive loading techniques, such as thin-film hydration or a modified passive loading approach, such as the proliposome method, which often result in vesicle heterogeneity due to variable encapsulation efficiencies and lamellarity. Upon incorporation of olive leaf extract, a discernible shift toward larger vesicle concentrations was observed, particularly in the AL and PG90 formulations. This shift may be attributed to the integration of olive bioactive compounds into the lipid bilayer or aqueous core, which can induce membrane changes or alter bilayer curvature [39,78,81]. Notably, the differences in the concentration increase between various samples indicate a formulation-dependent response to extract inclusion. The changes in the liposome vesicle concentration (as well as alterations of liposome size shown in the dynamic light scattering method) further corroborate the structural modifications induced by the extract. While all plain liposomes exhibited comparable concentrations (2.00–2.57 × 10^12^ particles/mL), extract-loaded AL and PG90 liposomal formulations showed significantly higher concentrations (6.87 × 10^12^ and 6.80 × 10^12^ particles/mL, respectively). This suggests that the incorporation of extracts in these systems promotes vesicle formation or stability during the preparation process. Demir et al.’s study [114] also demonstrated that the addition of the extract resulted in increased stability of the formulation compared to plain liposomes. Namely, improved colloidal stability provides reduced aggregation, thus maintaining a higher concentration of liposomes, i.e., a larger number of particles [115]. Conversely, the extract-loaded PH90 liposomal preparation demonstrated only a modest increase in particle concentration (2.70 × 10^12^ particles/mL), implying that the extract–lipid interaction may be less favorable in this composition, potentially due to differences in lipid phase behavior or bilayer packing density. According to the literature data, saturated PH90 showed very low loading rates and the absence of the influence of the encapsulated compound on the vesicle size compared to the unsaturated lipids [116,117]. These findings suggest that PH90 may interact poorly with certain compounds and does not promote significant extract-induced vesicle formation or higher count. Namely, the NTA data confirm that vesicle concentration is modulated by extract inclusion in a lipid-type-dependent manner, which could critically influence the biological behavior, release kinetics, and stability of the liposomal formulations. These observations are crucial for dose standardization in liposomal formulations, as vesicle count directly impacts the amount of encapsulated bioactive delivered per unit volume. The ability of unsaturated phospholipids (such as AL and PG90) to form higher numbers of vesicles upon extract loading is consistent with prior findings showing greater membrane fluidity and flexibility in unsaturated systems, which favor vesicle formation and encapsulation efficiency [71,72,73,74]. Future studies exploring the thermodynamic behavior of these lipid systems could further clarify the mechanisms driving these differences and optimize formulation strategies for targeted delivery applications.

The apparent discrepancies between the size values obtained by DLS and NTA can be explained by the fundamental differences in their measurement principles. DLS provides an intensity-weighted hydrodynamic diameter based on fluctuations in scattered light from the entire particle population, which inherently favors larger particles and may overestimate size in heterogeneous samples. In contrast, NTA tracks individual particle movements to generate a number-weighted distribution with higher resolution for polydisperse samples. Therefore, the larger sizes observed by DLS likely reflect the influence of a small fraction of larger vesicles or aggregates, while NTA more accurately represents the predominant particle population. These complementary results are consistent with the known methodological bias of DLS toward larger particles.

### 2.6. Transmission Electron Microscopy of Olive Leaf Extract-Loaded Liposomes

**Figure 5:**
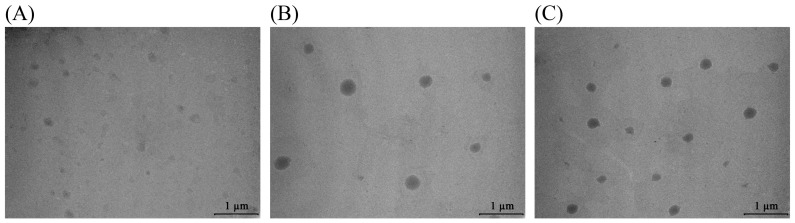
Representative transmission electron microscopy images of olive leaf extract-loaded liposomes: (**A**) liposomes prepared using phospholipids from producer Avanti (AL), (**B**) liposomes prepared using granulated phospholipids from producer Lipoid (PG90), and (**C**) liposomes prepared using hydrogenated phospholipids from producer Lipoid (PH90).

TEM analysis of olive leaf extract-loaded liposomes (AL, PG90, and PH90 formulations) demonstrated well-defined vesicles with intact structural features (Figure 5).

TEM images (Figure 5) reveal that liposomes prepared with AL phospholipids are the smallest vesicles with a higher heterogeneity, as already shown in the photon correlation spectroscopy measurement (Section 3.4). Those prepared using granulated phospholipids (PG90) show noticeably higher size and probably the occurrence of multilamellar structures, with well-defined spherical morphology. Liposomes based on hydrogenated phospholipids (PH90) are also larger, often with thicker bilayers or multiple concentric lamellae. These morphological differences are consistent with literature reports showing that higher lipid saturation (such as hydrogenated phospholipids) leads to increased bilayer rigidity, reduced curvature, and thus formation of larger vesicles [71]. The presence of the olive leaf extract may also influence morphology by inserting into or interacting with the lipid bilayer; however, the pronounced differences among AL, PG90, and PH90 are most likely driven by the lipid backbone saturation, purity, and the ability of lipids to pack tightly or allow bilayer bending, as shown in studies of hydrogenated vs. unsaturated phospholipids [71,118,119].

### 2.7. Rheological Characteristics of Developed Liposomes

The rheological characteristics of both blank and liposomes with extract were assessed immediately after preparation and following 60 days of refrigerated storage by measuring viscosity, surface tension, and density. The corresponding results are summarized in Table 3.

The viscosity of liposomal formulations differed significantly depending on the phospholipid composition (Table 3). On the 1st day, among plain liposomal formulations, PH90-based liposomes exhibited the highest viscosity (3.07 mPa·s), followed by PG90 (2.68 mPa·s) and AL (2.63 mPa·s). This pattern that different types of phospholipids significantly affected the mentioned parameter can be attributed to the higher degree of saturation and longer fatty acid chains in PH90, which promote tighter lipid packing and a more rigid bilayer structure, resulting in increased viscosity, while AL and PG90 liposomes contain unsaturated phospholipids with greater bilayer fluidity and disorder, contributing to reduced viscosity [74,120]. These findings align with previous reports indicating that bilayer rigidity and phase transition behavior directly influence the rheological properties of liposomal dispersions [76]. As shown in Table 3, the viscosity values of all unloaded liposomes were significantly lower than those of the liposomes loaded with olive leaf extract. Incorporation of the extract into the liposomal formulations resulted in an increase in viscosity from approximately 2.6 mPa·s to around 6 mPa·s (for AL and PG90 liposomes) and from 3.57 mPa·s to 4.49 mPa·s (for PH90 liposomes). Namely, the incorporation of the extract significantly increased the viscosity of all liposomal systems, regardless of phospholipid type; therefore, on the 1st day, extract-loaded PG90 liposomes exhibited the highest viscosity (6.78 mPa·s), followed by AL liposomes with extract (6.04 mPa·s) and PH90 liposomes with extract (4.49 mPa·s). This rise in viscosity may be attributed to the interaction of phenolic compounds from the extract with the lipid bilayers, which could promote bilayer stiffening or induce partial cross-linking within the vesicle matrix. Furthermore, the extract may increase the internal content viscosity or induce slight aggregation, contributing to higher resistance to flow. These findings support the notion that bioactive incorporation can significantly modify the physical properties of liposomal systems [121]. The viscosity of the prepared liposomes remained within a narrow range, as anticipated, since liposomal flow behavior is primarily influenced by temperature [122]. Shashidhar and Manohar noted that higher viscosity in liposomal systems typically reflects the presence of smaller liposome particles [79]. Accordingly, the olive extract-loaded liposomes exhibited significantly smaller diameters compared to the unloaded counterparts (Figure 3A) and also showed higher viscosity values (Table 3). Furthermore, PH90 liposomes with extract had larger particles (Figure 3A) and lower viscosity (Table 3) than AL and PG90 liposomes with extract. Nevertheless, previous findings have also shown that an increase in liposome diameter can trigger an increase in viscosity values. In this case, higher viscosity of the liposome system is caused by the establishment of complex linkage and crystallinity of particles in the central core due to their solid character [123]. These results emphasize the dual influence of both phospholipid composition and extract incorporation on the rheological behavior of liposomal systems, which can be crucial for optimizing formulation performance in topical or oral delivery routes. Namely, increasing bilayer rigidity through lipid saturation or bioactive loading leads to higher viscosities due to restricted vesicle mobility and enhanced inter-vesicular interactions. Moreover, controlling viscosity is not only important for processing and stability but also for ensuring appropriate release profiles, as more viscous systems may exhibit slower diffusion of encapsulated compounds, thereby prolonging bioactive availability.

After 60 days of storage, viscosity values changed for nearly all liposomal formulations, except for the plain AL liposomes and the extract-loaded PH90 liposomes (Table 3). Specifically, over 60 days, plain liposomes remained largely stable, with AL-based formulations showing negligible change (2.64 mPa·s), and PG90 and PH90 showing slight increases (3.05 mPa·s and 3.57 mPa·s, respectively). These modest changes may result from minor vesicle aggregation, slow fusion processes, or oxidative stress leading to altered bilayer organization [71,98]. Moreover, extract-loaded liposomes displayed formulation-dependent changes in viscosity during storage as well. AL-based liposomes with extract exhibited a marked increase in viscosity during storage (from 6.04 mPa·s to 7.70 mPa·s), suggesting progressive molecular interaction or bilayer tightening over time, possibly due to further integration of extract components, water loss, or hydrolysis [124]. PG90-based extract-loaded liposomes showed a notable decrease during 60 days (from 6.78 mPa·s to 5.44 mPa·s), which may reflect vesicle destabilization, partial leakage, or bilayer disruption [80]. PH90-based extract-loaded liposomes remained relatively stable during storage in the refrigerator, i.e., the viscosity varied from 4.49 mPa·s to 4.36 mPa·s, likely due to the high membrane integrity provided by saturated phospholipids [125]. These findings demonstrate that both the lipid matrix and the presence of bioactive compounds significantly influence the rheological behavior and long-term stability of liposomal systems. Such variations in viscosity over time are critical for predicting the shelf-life and handling properties of liposomal formulations, especially for applications requiring consistent flow behavior and dosing accuracy. The long-term stability of liposome viscosity is closely tied to lipid composition and storage conditions, with saturated lipids providing superior structural retention. These results suggest that choosing the appropriate phospholipid type not only affects initial formulation properties but also plays a key role in preserving functional characteristics during extended storage.

The surface tension measured immediately after liposome formation amounted to 26.4 mN/m for plain AL liposomes, 15.6 mN/m for plain PG90 liposomes, and 18.3 mN/m for plain PH90 liposomes (Table 3). The type of phospholipid used had a pronounced effect on the surface tension of plain liposomes. The liposomes prepared with PG90 exhibited the lowest surface tension, and these differences can be attributed to the degree of saturation and head group composition of the phospholipids. PG90 contains unsaturated phospholipids with a more fluid bilayer structure, allowing greater interfacial rearrangement and consequently reducing surface tension [73,125]. On the other hand, PH90 is fully saturated and forms more rigid bilayers with reduced dynamic flexibility at the interface, while AL contains both saturated and unsaturated lipids but with different proportions and origins, resulting in intermediate interfacial behavior [126,127]. These results are in agreement with previous findings, showing that surface tension correlates inversely with bilayer fluidity and interfacial lipid mobility [71]. Since plain PH90 liposomes had a large amount of foam, their significantly lower surface tension than that of plain AL liposomes can be caused by bubbles, which may disrupt the system of hydrogen bonds at the surface of the liquid [128]. The values of surface tension were significantly higher after the addition of olive extract: 29.6 mN/m for AL liposomes, 27.2 mN/m for PG90 liposomes, and 37.3 mN/m for PH90 liposomes (Table 3). Namely, the incorporation of the extract statistically significantly increased the surface tension of all liposomal types. In the case of augmented lipid packing (a smaller amount of interfacial water molecules), higher values of surface tension occur [129]. Hence, polyphenols from the extract can be incorporated between phospholipids within the liposome bilayer, creating smaller vesicles (as shown in Figure 3A) and explaining the higher values of surface tension of olive extract-loaded liposomes in comparison to their unloaded counterparts. The presence of plant extract (particularly rich in phenolic compounds) or plant-origin compounds can modify the surface activity of the system by interacting with the polar head groups and altering the bilayer packing and orientation at the interface [130,131]. Phenolics may either intercalate into the lipid membrane or adsorb to the surface, leading to increased intermolecular forces and reduced interfacial freedom, thereby elevating surface tension [121]. Similar effects have been reported with other polyphenol-loaded liposomes, where extract incorporation increased interfacial rigidity and disrupted surface packing. For example, Fathi-Azarbayjani et al. [132] also showed that an increase in the concentration of encapsulated compounds, without changing the concentrations of lipids/sterols, caused an increment in the liposome surface tension. As can be seen from Table 3, different phospholipids and entrapped bioactive molecules significantly influenced the surface tension of the liposome system, which agrees with the literature data where properties and content of phospholipids and encapsulated ingredients affected the mentioned variable [132], as in the case of viscosity.

Over 60 days, plain liposomes exhibited variable trends in surface tension (Table 3). PG90- and PH90-based plain liposomes showed minor changes (remaining around 16.0 mN/m and 20.3 mN/m, respectively), suggesting relatively stable interfacial organization in these systems. However, plain AL liposomes experienced a marked reduction (from 26.4 mN/m to 17.9 mN/m), which could be attributed to lipid degradation or reorientation of head groups, leading to altered interfacial properties [39,131]. Extract-loaded liposomes, on the other hand, largely maintained their surface tension values over the 60 days. AL and PG90 extract-loaded formulations showed only slight changes (from 29.6 mN/m to 28.8 mN/m and 27.2 mN/m to 28.1 mN/m, respectively), indicating good interfacial stability. Notably, extract-loaded PH90 liposomes showed a slight increase (from 37.3 mN/m to 39.4 mN/m), suggesting further surface structuring or bilayer condensation during storage. The stabilizing effect of the extract could be due to persistent binding of phenolic compounds to the lipid head groups or their antioxidant role in preserving bilayer integrity, as shown in previous studies [59,109]. Collectively, these findings demonstrate that both lipid composition and extract presence significantly influence interfacial properties, and extract-loaded liposomes show better surface tension stability during long-term storage. These interfacial stability trends are especially important for applications where surface behavior influences bioavailability, such as mucosal delivery or emulsification in food and cosmetic systems. It should also be highlighted that phenolic–phospholipid interactions can lead to the formation of more ordered interfacial films, contributing to prolonged stability and enhanced protection against environmental stressors. Therefore, maintaining or even slightly increasing surface tension in extract-loaded liposomes over time supports the hypothesis that polyphenols contribute not only to bioactivity but also to structural stabilization of the liposomal membrane.

The density of the plain and olive extract-loaded liposomal particles was measured on the 1st and 60th days of storage (Table 3). On the 1st day, all plain liposomes displayed density values near that of pure water (~0.999–1.002 g/cm^3^), reflecting their aqueous dispersion and the relatively low mass contribution of the lipid bilayers. On the other hand, the density of plain AL and PG90 liposomes was the same (0.999 g/cm^3^), while PH90 liposomes showed a higher value of density (1.002 g/cm^3^) but without a statistically significant difference; thus, no specific trend in the influence of phospholipids on the density of liposomal formulation was observed. The addition of extract notably increased the density of the systems, most markedly in the PG90 liposomes population with extract (1.018 g/cm^3^), suggesting successful encapsulation of higher-molecular-weight or denser extract constituents within or associated with the lipid bilayers. The AL and PH90 extract-loaded systems also showed increased density (1.007 g/cm^3^ and 1.005 g/cm^3^, respectively), indicating some formulation-dependent interaction with the bioactive components, which is in agreement with the literature data [28]. The addition of olive extract to liposomal formulations increases their density through several tightly interwoven molecular mechanisms. Rich in polyphenolic compounds such as oleuropein, the extract interacts with lipid bilayers by integrating into the membrane structure, enhancing lipid packing and reducing membrane fluidity, which results in a denser organization. Moreover, the olive extract’s hydrophilic and amphiphilic constituents are efficiently encapsulated within the aqueous core or bilayer, increasing the internal mass and contributing to overall density [48].

Density measurements offer an indirect but informative view of liposomal structural integrity and potential compositional rearrangements during storage. Across the 60 days, liposomes exhibited subtle but interpretable trends in density values based on phospholipid composition and the presence of the olive leaf extract. After 60 days of storage, the plain liposomes maintained density values close to their initial state (~1.000–1.001 g/cm^3^), with only minimal deviations. This suggests that no major structural breakdown or lipid loss occurred in the unloaded systems, supporting their physical stability over time. Additionally, extract-loaded liposomes maintained higher densities than the plain controls, with slight decreases observed in PG90 liposomes (from 1.018 g/cm^3^ to 1.011 g/cm^3^). This minor reduction could be indicative of partial release or rearrangement of the encapsulated extract compounds or modest bilayer reorganization [133]. Interestingly, PG90 liposomes with extract remained the most dense throughout the study, possibly due to good structural integrity and minimal disruption by the extract, which may enhance the retention of encapsulated compounds [134]. Conversely, PH90- and AL-based systems exhibited slightly lower densities and marginally more fluctuation, which may reflect their greater bilayer dynamics and permeability [71,90]. This reduction may reflect differences in lipid packing, bilayer rigidity, or interaction with the encapsulated compounds. Hydrogenated phospholipids (such as PH90), due to their saturated nature, tend to form more rigid bilayers that could entrap less extract or water, thus lowering density. Similarly, the less compact structure of soy-derived phospholipids might contribute to lower mass per unit volume. From a statistical perspective, while some values shifted between groups (e.g., significant differences noted between PG90 and AL or PH90 liposomes), the overall density changes remained within ±0.02 g/cm^3^, suggesting that all systems retained general structural cohesion. This supports the utility of liposomes, particularly extract-loaded ones, for maintaining encapsulation over extended periods, with PG90-based formulation emerging as the most robust under the studied conditions. These findings are in line with the facts that denser liposomal systems often correlate with improved encapsulation efficiency and long-term retention of hydrophilic and amphiphilic bioactives. Furthermore, the relatively stable density values observed over storage support the structural resilience of the formulations, reinforcing the suitability of liposomes, particularly those based on PG90, as effective carriers for polyphenol-rich extracts. Future studies could combine density analysis with release kinetics and permeability assays to better understand how internal structural changes correlate with bioactive retention and delivery performance.

### 2.8. Release of Olive Leaf Extract Polyphenols Under Simulated Physiological Conditions

**Figure 6:**
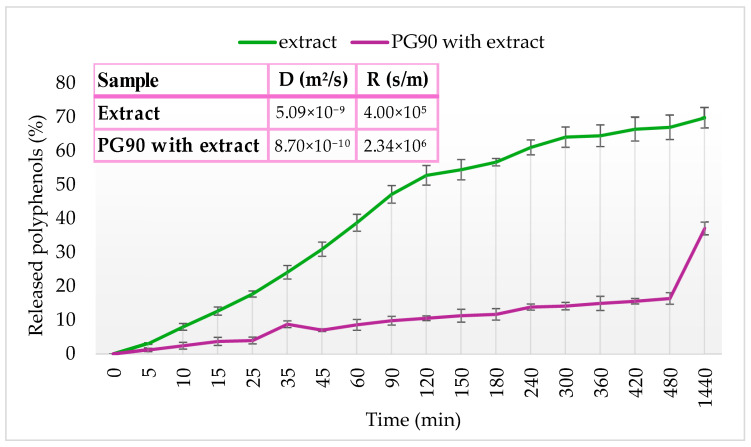
Polyphenol release curves from pure olive leaf extract and encapsulated liposomal formulation (PG90 liposomes) in simulated physiological conditions (phosphate-buffer saline, pH 7.4, 37 °C) over 24 h; PG90 liposomes were prepared using granulated phospholipids from producer Lipoid; D—diffusion coefficient and R—diffusion resistance.

The release behavior of polyphenols from both the pure olive leaf extract and selected encapsulated formulation (PG90 liposomes with extract, due to the highest EE of oleuropein as the most dominant compound of the extract, Table 2) was assessed under simulated physiological conditions (PBS, pH 7.4, 37 °C) and measured spectrophotometrically [135]. Polyphenol release was investigated using a Franz diffusion cell to evaluate the mass transfer resistance presented by the liposomal membrane. The release profiles are illustrated in Figure 6, where the release profiles are presented as the percentage of released polyphenols over time, monitored for 24 h. Analysis of the polyphenol release data allowed for the calculation of diffusion coefficients and diffusion resistances associated with the liposomal formulation; the methodology for these calculations is detailed in the Supplementary Material. The resulting diffusion coefficients and resistances are summarized in a table within Figure 6.

The release behavior of polyphenols from liposomes containing PG90 phospholipids was compared to that of the free olive leaf extract, which served as a control at the same polyphenol concentration used during liposome formulation. Only the liposome sample with the highest encapsulation efficiency was selected for the release study. As illustrated in Figure 6, polyphenols from the free olive leaf extract exhibited rapid diffusion, reaching peak concentrations in the acceptor compartment after approximately 240 min in the PBS medium. In contrast, the release from liposomal formulation was markedly slower, as anticipated. After 24 h, 69.8 ± 1.7% of polyphenols were released from the pure extract, compared to only 37.1 ± 1.9% from the olive leaf extract-loaded liposomes. These findings indicate that liposomes effectively retain polyphenols and are therefore promising candidates for sustained release applications [28,136,137]. To better understand the mass transport behavior of polyphenols from different formulations (extract and liposomes with extract), the diffusion coefficients (D) and corresponding diffusion resistances (R) were evaluated under simulated physiological conditions. The obtained values, as summarized in a table in Figure 6, clearly reflect the impact of liposomal encapsulation on the transport properties of polyphenols. For the free olive leaf extract, the diffusion coefficient was calculated to be 5.09 × 10^−9^ m^2^/s, with an associated resistance of 4.00 × 10^5^ s/m. These values indicate a relatively fast diffusion rate and low mass transfer resistance, consistent with the observed rapid release profile in the tested medium. The absence of a physical barrier (such as a lipid membrane) allows polyphenols to freely diffuse through the donor-acceptor system, resulting in quicker saturation of the acceptor compartment. In contrast, the olive polyphenol-loaded liposomal formulation exhibited a significantly lower diffusion coefficient of 8.70 × 10^−10^ m^2^/s and a correspondingly higher resistance of 2.34 × 10^6^ s/m. This substantial reduction in D and increase in R demonstrates the restrictive nature of the liposomal bilayer, which imposes a significant barrier to polyphenol diffusion. These values correlate well with the delayed and prolonged release profiles observed in the graphs of Figure 6. The lipid bilayer acts as a diffusion-limiting structure, reducing the mobility of encapsulated compounds and effectively modulating their release kinetics. The elevated resistance (R) in liposomes can be attributed to both the physical encapsulation of polyphenols within the vesicular structure and the potential reorganization or tightening of the lipid bilayer, which may undergo additional membrane stabilization or crosslinking [28,136,137,138]. This controlled release behavior is advantageous in pharmaceutical applications where a sustained delivery of active compounds is desired. Furthermore, the difference in D and R between the free extract and liposomal formulations underlines the importance of the delivery system in controlling bioactive release. While rapid diffusion (high D, low R) is suitable for immediate therapeutic effects, liposomal encapsulation (low D, high R) offers a more sustained, protective, and targeted release profile, reducing degradation and enhancing bioavailability in complex biological environments.

The liposomal encapsulation of olive leaf extract presents promising opportunities across several fields. In functional foods and nutraceuticals, liposomes can enhance the bioavailability and stability of polyphenols, enabling the development of value-added products with antioxidant and health-promoting benefits. Additionally, topical delivery systems may benefit from liposomal formulations by improving skin penetration and sustained release of active compounds for cosmetic and therapeutic applications. A recent study investigated the gastrointestinal stability of olive leaf phenolic compounds, which incorporated co-administration with inulin and microencapsulation techniques, by using in vitro digestion models [139]. The results revealed that both strategies significantly influenced the degradation profile of phenolics. Inulin enhanced the stability of these compounds by forming protective interactions under digestive conditions, particularly improving the gastric and intestinal resilience of oleuropein and modulating the breakdown of hydroxytyrosol precursors, favoring the preservation of its glucoside form. Microencapsulation further strengthened this protective effect, offering improved compound retention despite minor degradation in the gastric phase. Complementary studies by Paulo and Santos [140] and Flammini et al. [141] further support the value of microencapsulation in preserving olive-derived phenolics from environmental degradation and enabling their sustained release in enriched foods. Flammini et al. [141] demonstrated that microencapsulation of olive leaf extract influenced the release rate of phenolics depending on system pH. Together, these findings underscore the potential of tailored encapsulation approaches to improve the stability, delivery, and nutritional impact of olive leaf extracts in functional food applications. However, several challenges remain for industrial implementation, including scalability of liposome production, cost-effectiveness, long-term stability under varying storage conditions, and regulatory considerations related to food and pharmaceutical approvals. Addressing these hurdles will be critical to translating these promising laboratory findings into commercially viable products.

## 3. Materials and Methods

### 3.1. Chemicals

The following phospholipids were used for the liposome formation: (1) soy L-α-phosphatidylcholine (fatty flakes, purity >95%, Avanti Polar Lipids, Alabaster, AL, USA)—AL, (2) Phospholipon ^®^ 90 G (fatty flakes, ≥94%, soybean unsaturated diacyl-phosphatidylcholine, Lipoid GmbH, Ludwigshafen, Germany)—PG90, and (3) Phospolipon ^®^ 90 H (fatty powder, ≥ 90%, hydrogenated soybean phospholipids, Lipoid GmbH, Ludwigshafen, Germany)—PH90. Olive leaves were purchased from the Institute for Medicinal Plant Research “Dr Josif Pančić” (Belgrade, Serbia). Ethanol (96%, REAHEM D.O.O., Srbobran, Serbia) was used for the extract preparation. For the HPLC and GC-MS analyses, the following reagents and standards were employed: oleuropein (>95%), oleacein (90%), oleuropein aglycone (95%), hydroxytyrosol (95%), tyrosol (98%), luteolin-7-O-glucoside (94%), maslinic acid (>95%), oleanolic acid (>95%), and quercitrin (98%) from LGC Standards (Teddington, UK); ligstroside (96%) from Toronto Research Chemicals (Toronto, ON, Canada); apigenin-7-O-glucoside (93%, Sigma-Aldrich, Strasbourg, France); chlorogenic acid and quercetin (Sigma-Aldrich, St. Louis, MO, USA); and trans-androsterone (>97%, Fluka, Buchs, Switzerland). Ethanol (HPLC grade, JT Baker, Phillipsburg, NJ, USA), acetic acid (Merck KGaA, Darmstadt, Germany), ultrapure water (LiChrosolv^®^ grade, Merck KGaA, Darmstadt, Germany), and acetonitrile (ACN, HPLC grade, Merck KGaA, Darmstadt, Germany) were used as solvents for preparing the mobile phase. Ethyl acetate and BSTFA (N,O-bis(trimethylsilyl)trifluoroacetamide) were from Merck KGaA (Darmstadt, Germany). ABTS (2,2′-azino-bis (3-ethylbenzothiazoline-6-sulphonic acid)), DPPH (2,2-diphenyl-1-picrylhydrazyl), paraformaldehyde, glutaraldehyde, and ascorbic acid were obtained from Sigma Aldrich (Steinheim, Germany), while potassium persulfate (K_2_S_2_O_8_) was obtained from Centrohem (Stara Pazova, Serbia). PBS (phosphate-buffered saline, Sigma Aldrich, Darmstadt, Germany, 10 mM, pH 7.4) was used for the release kinetic study.

### 3.2. Olive Leaf Extract Preparation

Olive extract was prepared using 16 g of the intensively grinded leaves (particle size lower than 0.3 mm) and 80% *v*/*v* ethanol (20 mL) in an ultrasound extraction (ultrasound bath SONOREX™ SUPER, BANDELIN electronic, Berlin, Germany) for 30 min. The process was repeated three times to achieve the maximal utilization of the plant’s raw materials. The sample was filtered through filter paper, and ethanol was evaporated in the Hei-VAP (Heidolph, Heidelberg, Germany) at 50 °C in a vacuum for 30 min. The obtained dried extract was stored at 4 °C until further analysis.

### 3.3. Olive Leaf Extract-Loaded Liposome Preparation

The liposomes with ethanol olive leaf extract were formed in the proliposome procedure [34]. All liposomal formulations were prepared using the same phospholipid concentration and hydration method to ensure comparability. No cholesterol or additional membrane stabilizers were included in any of the formulations to ensure proper and unbiased comparison across the different phospholipid types. The dried olive extract was dissolved in 80% ethanol to achieve a concentration of 100 mg/mL. A total volume of 8 mL of the prepared extract solution was combined with 2 g of selected phospholipids (AL, PG90, or PH90) and heated at 60 °C for 30 min to facilitate ethanol evaporation and promote the formation of a uniform lipid mixture. Subsequently, 40 mL of aqueous medium (ultrapure water) was gradually introduced in small aliquots, with continuous stirring at 1000 rpm for 2 h at room temperature. Since, after ethanol evaporation, 40 mL of aqueous medium was gradually added to the lipid-extract mixture, the final liposomal dispersion had a total volume of approximately 40 mL, containing 20 mg/mL of olive leaf extract and 50 mg/mL of phospholipids. The liposomes were prepared using a conventional hydration method, where the lipid mixture was hydrated directly using a micropipette and tip. All formulations were prepared using identical concentrations of extract and lipids, and the procedure provided comparability. Control liposomes (blank formulations) were prepared using the same procedure, with 8 mL of 80% *v*/*v* ethanol added in place of the olive leaf extract. All liposomal dispersions were stored at 4 °C until further characterization. The solution, in a volume of 8 mL, was mixed with 2 g of phospholipids (AL, PG90, or PH90) and heated to 60 °C for 30 min to evaporate ethanol and obtain a homogeneous.

### 3.4. HPLC and GC-MS Chemical Composition Analysis and Encapsulation Efficiency

Pure olive leaf extract and its liposomal encapsulates were subjected to HPLC and GC-MS analyses. Before analysis, to separate the non-encapsulated fraction of olive extract from the liposomes with encapsulated compounds, the centrifugation of liposomes with extract was performed at 4 °C and 17,500 rpm for 45 min in a Thermo Scientific Sorval WX Ultra series ultracentrifuge (Thermo Fisher Scientific, Waltham, MA, USA). The amount of individual compounds was determined in the supernatants and centrifuged liposomes, where phospholipids were dissolved using 96% ethanol. The pellet obtained after centrifugation was dissolved in 96% ethanol, and the entire volume was used for HPLC analysis to ensure that all analytes released from the liposomes were quantitatively assessed. Although formal validation of the dissolution step was not conducted, complete volume analysis minimized the risk of analyte loss. The analyses were performed on the 1st and 60th days of storage.

HPLC analysis of secoiridoids, flavonoids, flavonoid glycosides, and simple phenols was performed using an Agilent 1100 apparatus (Agilent Technologies, Santa Clara, CA, USA). A Zorbax Eclipse XDB-C18 column (150 × 4.6 mm, particle size of 5 µm) (Agilent Technologies, Santa Clara, CA, USA) was used. Identification was performed using a DAD detector (photodiode array detector) at 250 nm (oleuropein, oleuropein aglycone, oleacein, ligstroside, quercetin, and quercitrin), 330 nm (apigenin-7-O-glucoside and chlorogenic acid), and 350 nm (luteolin 7-O-glucoside). The test was performed with an appropriate gradient flow of the mobile phase (0.8 mL/min, 25 °C); mobile phase A: 2.5% acetic acid in water, and mobile phase B: acetonitrile (ACN). The gradient flow was as follows: 0 min 95% A, 5% B; 0–20 min 75% A, 25% B; 20–40 min 50% A, 50% B; 40–50 min 20% A, 80% B; 50–60 min 95% A, 5% B, and 65 min 95% A, 5% B. The volume of injection was 5 µL. The compounds were identified by comparing their UV spectra and retention times with those of chemical standard substances, while quantification was performed using external calibration curves obtained from standard solutions analyzed under the same experimental conditions. The HPLC method was developed based on a previously described procedure [12] and validated by evaluating its selectivity, linearity, precision, reproducibility, accuracy, and recovery (Supplementary Materials, Table S1).

GC-MS method, as previously described [142], was used for the quantification of oleanolic and maslinic acids, and was carried out using an Agilent Technologies 6890 N gas chromatograph equipped with an Agilent 7683 autosampler and coupled to an Agilent 5973 mass selective detector (MSD) (Agilent Technologies, Santa Clara, CA, USA) operating with electron impact (EI) ionization (70 eV). In brief, 200 µL of each sample was mixed with 100 µL of internal standard solution (trans-androsterone, 100 µg/mL in methanol), diluted with water, and extracted with 5 mL of ethyl acetate for 5 min at room temperature. The organic phase was evaporated to dryness, and the residue was weighed and reconstituted in 100 µL of BSTFA. Silylation was performed at 60 °C for 20 min. A volume of 3 µL of the derivatized sample was injected into the GC–MS system. Chromatographic separation was achieved using a DB–5MS capillary column (30 m × 0.25 mm i.d., 0.25 µm film thickness; J&W Scientific, Folsom, CA, USA). The oven temperature program was as follows: initially held at 250 °C for 1 min, ramped at 22 °C/min to 280 °C, and maintained at 280 °C for 34.00 min. Total run time was 36.6 min. The injector temperature was set at 250 °C, with a 1.0 min splitless injection (purge off), using helium as the carrier gas at a constant flow rate of 54.1 mL/min. The transfer line and detector temperatures were set at 280 °C. Mass spectrometry conditions included EI ionization at 70 eV, operating in selected ion monitoring (SIM) mode for targeted quantification (*m*/*z* = 73). Quantification was performed using the ratio of peak areas of the target analytes to the internal standard (trans-androsterone) and applying a calibration curve constructed with chemical standard substances analyzed under the same experimental conditions.

### 3.5. Fourier Transform Infrared Spectroscopy

FT-IR analysis was used to characterize the chemical composition of three different olive leaf extract-loaded liposomes, as well as pure olive leaf extract and plain liposomes. Spectra were recorded using the ATR mode on a Nicolet IS35 FTIR-ATR spectrometer (Thermo Fisher Scientific, Uppsala, Sweden) over a wavelength range of 500–4000 cm^−1^. As the analytical device required water-free samples, liposomal formulations were subjected to freeze-drying using an Alpha 2–4 LSCplus lyophilizer (Christ, Osterode am Harz, Germany). Before lyophilization, the liposomal samples, after centrifugation to remove the non-encapsulated fraction of the olive leaf extract, were frozen and then dried under vacuum conditions (0.011 mbar) at −75 °C for 24 h. The same procedure was performed for the plain liposomes, but without the centrifugation step.

### 3.6. Evaluation of the Antioxidant Activity of Liposomes

The antioxidant properties of the prepared liposomal formulations with olive leaf extract and pure extract (diluted to achieve the same concentration as in the case of extra-loaded liposomal formulations) were assessed employing two distinct assays: ABTS and DPPH radical scavenging tests. Additionally, the antioxidant efficacy of the plain liposomes was examined as well.

#### 3.6.1. ABTS Radical Scavenging Assay

The ability of developed liposomes and olive leaf extract to scavenge ABTS radicals was determined following the procedure adapted from Mohammadi et al. [143] with minor modifications. Briefly, ABTS solution was prepared by mixing 0.019 g of ABTS powder in 5 mL of water, followed by the addition of 88 µL potassium persulfate. This mixture was allowed to react for 24 h at 4 °C. The resulting ABTS^•+^ solution was then diluted with ethanol until an absorbance of approximately 0.700 at 734 nm was achieved. The assay was performed by mixing 2 mL of the prepared ABTS^•+^ working solution with 20 µL of the liposomal formulation, and absorbance was measured using a UV-1800 spectrophotometer (Shimadzu, Kyoto, Japan). After a 6-min incubation period, the absorbance was recorded, and the radical scavenging activity was calculated as follows (Equation (1)):

(1) $$\%\;inhibition\;=\;100\;\times \;\frac{(A_{0}\;-\;A_{x})\;}{A_{0}}$$

where A_0_ represents the absorbance of the ABTS^•+^ solution alone, and A_x_ is the absorbance after the addition of the liposomes/extract. Ascorbic acid (400 µg/mL) served as the positive control. The results are presented as the percentage of the inhibition.

#### 3.6.2. DPPH Radical Scavenging Assay

The antioxidant potential of liposomal formulations and extract was further evaluated based on their capacity to donate hydrogen atoms to stable DPPH^•^ radicals [143]. Different concentrations of the liposomal samples (200 µL) were mixed with 2 mL of ethanol-based DPPH^•^ solution, adjusted to an absorbance near 0.800 at 517 nm. Following a 20-min incubation, the absorbance was measured, and the percentage inhibition was calculated using the formula (Equation (2)):

(2) $$\%\;inhibition\;=\;100\;\times \;\frac{(A_{0}\;-\;A_{x})\;}{A_{0}}$$

where A_0_ denotes the absorbance of the control DPPH^•^ solution, and A_x_ is the absorbance in the presence of the liposomes/extract. Ascorbic acid (400 µg/mL) was used as a reference antioxidant. The results are presented as the percentage of the inhibition.

### 3.7. Monitoring of Liposome Stability

The particle size, polydispersity index (PdI), electrophoretic mobility, and zeta potential of both blank and olive extract-loaded liposomal formulations were evaluated over a 60-day storage period at 4 °C. The liposomal formulations were stored at 4 °C to reflect the intended application conditions, as their high water content prevents safe storage at room temperature, and the formulations are designed for direct use in liquid form for future animal studies without exposure to elevated industrial temperatures. These parameters were assessed using dynamic light scattering (DLS), based on photon correlation spectroscopy, with a Zetasizer Nano Series instrument (Malvern Instruments, Malvern, UK). Measurements were carried out at regular intervals (days 7, 14, 21, 28, and 60) with each sample analyzed in triplicate at 25 °C. Before analysis, the samples were diluted 1:500 (*v*/*v*), and 1 mL of the diluted dispersion was used per measurement.

### 3.8. Nanoparticle Tracking Analysis

The concentration and size distribution of the liposome preparations (unloaded and olive extract-loaded liposomes) were evaluated using the ZetaView Quatt PMX-430 nanoparticle tracking analyzer (NTA), operated with ZetaView software version 8.05.16 SP3 (Particle Metrix, Inning am Ammersee, Germany). Before measurement, the system performed an automatic cell check, followed by camera and laser alignment. Instrument calibration was confirmed using 100 nm polystyrene beads, in accordance with the manufacturer’s instructions. Liposome preparations were diluted in deionized water to ensure an optimal number of particles per frame. A cleaning step was included between individual measurements. Analyses were carried out in light scatter mode (LSM) using a 488 nm blue laser. Video recording parameters were set to a shutter speed of 100 and a frame rate of 30 frames per cycle, with a sensitivity level of 78. Post-capture analysis settings included a minimum area of 10, a maximum area of 1000, and a minimum brightness threshold of 30. Each sample was measured in triplicate at up to 11 positions.

### 3.9. Transmission Electron Microscopy

For morphological analysis, 10 µL of each liposomal formulation was placed onto 200-mesh formvar-coated copper grids and left to adsorb at room temperature for 30 min. The samples were then fixed by floating the grids on a 2% paraformaldehyde solution for 10 min, followed by three rinses with ultrapure water. A secondary fixation step was carried out using 2.5% glutaraldehyde for 5 min, after which the grids were rinsed again with ultrapure water for 5 min. Finally, the samples were air-dried at room temperature and imaged using a Philips CM12 transmission electron microscope (Philips, Eindhoven, The Netherlands).

### 3.10. Examination of Rheological Characteristics

Plain and extract-loaded liposomal formulations (6.7 mL) were placed in a chamber equipped with a spindle (Rotavisc lo-vi, IKA, Staufen, Germany) to assess their viscosity at 25 °C, using a rotational speed of 200 rpm. Each measurement was conducted in triplicate and repeated following 60 days of storage. Additionally, the surface tension and density of both unloaded and extract-loaded liposomes (20 mL) were determined in a glass beaker using a Force Tensiometer K20 (KRÜSS, Hamburg, Germany). Density measurements were performed via the immersed body method, whereas surface tension was evaluated using the Wilhelmy plate technique. These assessments were also performed in triplicate and repeated after 60 days of storage.

### 3.11. In Vitro Release Kinetics Using Franz Diffusion Cell

The release kinetics of polyphenols from the pure olive leaf extract and selected liposomal formulation were evaluated using a Franz diffusion cell system (PermeGear, Inc., Hellertown, PA, USA). The experiments simulated physiological conditions at 37 °C, employing an acetate cellulose membrane to separate the donor and receptor compartments, while PBS was used as the receptor medium [133]. The donor compartment was filled with 2 mL of either the extract (dissolved to achieve the same concentration as in the liposomes) or liposomal sample, applied directly onto the membrane. The receptor compartment was filled with PBS, maintained at 37 °C with continuous magnetic stirring at 850 rpm using a water-jacketed system and a peristaltic pump. Polyphenol release into the receptor medium was monitored over 24 h. At defined time intervals, 500 µL of aliquots was withdrawn from the receptor compartment, and the concentration of released polyphenols was measured spectrophotometrically at 270 nm.

### 3.12. Statistical Data Processing

All statistical analyses were performed using STATISTICA 7.0 software (StatSoft Inc., Tulsa, OK, USA). Data are presented as mean ± standard deviation from three independent experiments (n = 3). Before applying parametric tests, data were assessed for normality using the Shapiro–Wilk test and for homogeneity of variance using Levene’s test. Upon confirming these assumptions, one-way analysis of variance (ANOVA) was conducted to determine statistically significant differences between groups. For post hoc multiple comparisons, Duncan’s multiple range test was applied to identify pairwise differences among group means. Statistical significance was set at *p* < 0.05. This approach allowed for robust comparison between liposomal formulations across all measured parameters, including EE, antioxidant activity, particle size, PdI, zeta potential, and electrophoretic mobility.

## 4. Conclusions

This study focused on determining the formulation and thorough physicochemical and morphological characterization and release kinetics of liposomal delivery systems developed to enhance the solubility and stability of bioactive compounds extracted from olive leaf. The chemical composition analysis, conducted via HPLC and GC-MS, confirmed that oleuropein is the dominant compound in the ethanolic olive leaf extract. Additionally, the analysis confirmed that oleuropein is efficiently encapsulated in all liposomal types, with PG90 liposomes achieving the highest encapsulation efficiency. Triterpenes such as oleanolic and maslinic acids showed the highest retention in PH90 liposomes, indicating that saturated phospholipids favor lipophilic compound encapsulation. Flavonoid glycosides like luteolin and apigenin were most effectively encapsulated in AL and PG90 liposomes, demonstrating that unsaturated phospholipids better accommodate polar compounds. FT-IR analysis indicated that liposomes containing non-hydrogenated phospholipids (AL and PG90 samples) show broader CH_2_ stretching bands, greater shifts in the O–H and C=O regions, and more pronounced changes in phosphate headgroup vibrations, i.e., signs of stronger interaction with olive leaf extract, whereas hydrogenated phospholipids (PH90 formulation) produce more defined, less shifted bands, consistent with a more ordered, less interactive bilayer. The consistently high radical-scavenging capacity of olive leaf extract-loaded liposomes, contrasted with the minimal activity of empty carriers, indicates that the liposomal matrix functions solely as a protective delivery vehicle, ensuring retention of the extract’s phenolic-derived antioxidant potential and supporting its applicability in pharmaceutical formulations aimed at mitigating oxidative stress. Stability analysis revealed that extract-loaded liposomes had smaller particle sizes and more negative zeta potential than unloaded ones, suggesting improved colloidal stability due to extract incorporation. Over 60 days, PG90 liposomes with extract maintained consistent electrophoretic mobility and zeta potential, indicating long-term stability. Nanoparticle tracking analysis revealed that extract-loaded liposomes had narrower size distributions and higher particle concentrations, indicating that they exhibited structural uniformity. The lipid composition, particularly saturation level and purity play a critical role in determining liposome morphology, with hydrogenated phospholipids promoting the formation of larger, multilamellar vesicles due to increased bilayer rigidity and reduced curvature. Rheological evaluations demonstrated that extract-loaded liposomes had higher viscosity and surface tension compared to plain liposomes. Finally, density measurements indicated that extract incorporation increased liposome density across all formulations, reflecting tighter lipid packing and enhanced structural integrity. Based on the stability and encapsulation profiles observed, PG90 liposomes are preferred for oral delivery due to their superior EE and long-term colloidal stability. In contrast, PH90 liposomes, with enhanced retention of lipophilic triterpenes, may be more suitable for topical applications targeting skin-related oxidative stress. Collectively, these findings highlight the significance of phospholipid selection in tailoring liposomal systems for optimal encapsulation, stability, and delivery of olive leaf bioactives, which is crucial for their future biological efficacy and applications. The markedly lower diffusion coefficient and higher resistance observed in liposomal formulations highlight the role of the lipid bilayer as a diffusion-limiting barrier, effectively modulating polyphenol release. These findings confirm that liposomes are suitable delivery systems for sustained and controlled release applications, offering enhanced protection and bioavailability of encapsulated compounds. A comprehensive thermodynamic analysis of developed liposomal formulations, including parameters such as Gibbs free energy and temperature-dependent stability, is planned for future experiments to further understand their physicochemical behavior and stability under application-relevant conditions. Regarding the degradation of oleuropein under microbial metabolism, particularly in the colon, where gut bacteria such as *Lactobacillus* and *Bifidobacterium* contribute to the enzymatic transformation of oleuropein into bioactive metabolites, and given the complexity of microbial testing and its dependence on advanced in vitro or in vivo gut models, this important direction will be considered in future experiments to complement the initial formulation work presented here. Furthermore, recognizing the limitations of in vitro testing alone, future studies will include in vivo investigations to evaluate the bioavailability and pharmacodynamic performance of the optimized liposomal formulation under physiological conditions, in accordance with ethical regulations and following the principles of the 3Rs (Replacement, Reduction, and Refinement) to ensure responsible use of animal models.

## Figures and Tables

**Table 1:** Quantitative analysis of olive leaf extract and extract-loaded liposomes.

Sample		Olive Leaf Extract	Extract-Loaded Liposomes		
			AL	PG90	PH90
Class of Compounds	Compound	µg/mg Dry Extract			
Secoiridoids	Oleuropein	111.8 ± 10.85 ^a,^*	98.28 ± 3.50 ^a^	85.75 ± 4.18 ^b^	87.18 ± 2.18 ^d^
	Oleacein	3.75 ± 0.24 ^b^	4.23 ± 0.15 ^a^	4.25 ± 0.15 ^a^	4.03 ± 0.10 ^ab^
	Ligstroside	1.06 ± 0.14 ^ab^	0.98 ± 0.06 ^b^	0.99 ± 0.04 ^b^	1.28 ± 0.20 ^a^
	Oleuropein aglycone	0.43 ± 0.08 ^a^	0.24 ± 0.02 ^b^	0.36 ± 0.05 ^a^	0.31 ± 0.03 ^a^
Pentacyclic triterpenes	Oleanolic acid	42.88 ± 3.10 ^a^	15.61 ± 1.10 ^c^	20.94 ± 1.6 ^b^	43.28 ± 2.10 ^a^
	Maslinic acid	5.76 ± 0.38 ^a^	0.18 ± 0.03 ^d^	0.31 ± 0.05 ^c^	3.11 ± 0.20 ^b^
Flavonoids and flavonoid glycosides	Luteolin 7-O-glucoside	9.84 ± 1.21 ^a^	7.82 ± 0.60 ^b^	7.04 ± 0.15 ^c^	7.74 ± 0.31 ^b^
	Apigenin- 7-O-glucoside	5.21 ± 0.65 ^a^	3.94 ± 0.80 ^ab^	3.75 ± 0.20 ^b^	4.40 ± 0.18 ^a^
	Quercetin	0.63 ± 0.08 ^a^	0.50 ± 0.10 ^ab^	0.45 ± 0.05 ^b^	0.49 ± 0.07 ^ab^
	Quercitrin	0.18 ± 0.01 ^ab^	0.20 ± 0.01 ^a^	0.16 ± 0.03 ^ab^	0.17 ± 0.01 ^b^
Simple phenols	Hydroxytyrosol	1.56 ± 0.28 ^ab^	2.15 ± 0.35 ^a^	1.90 ± 0.10 ^a^	1.60 ± 0.05 ^b^
	Chlorogenic acid	0.14 ± 0.03 ^a^	0.08 ± 0.01 ^b^	0.05 ± 0.01 ^c^	0.09 ± 0.02 ^ab^

* The results are expressed as the mean value ± standard deviation based on three independent replicates. AL—liposomes prepared using phospholipids from producer Avanti, PG90—liposomes prepared using granulated phospholipids from producer Lipoid, PH90—liposomes prepared using hydrogenated phospholipids from producer Lipoid. Identical letters within each row indicate no statistically significant differences (for each compound individually) based on one-way ANOVA followed by Duncan’s post hoc test, with significance set at *p* > 0.05 (n = 3).

**Table 2:** Quantitative analysis of encapsulated extract fraction within liposomes and encapsulation efficiency.

Sample		Encapsulated Extract Fraction in Liposomes					
		AL		PG90		PH90	
Class of Compounds	Compound	µg/mg d.e.	EE (%)	µg/mg d.e.	EE (%)	µg/mg d.e.	EE (%)
Secoiridoids	Oleuropein	71.65 ± 6.64 ^a,^*	72.90 ± 2.76 ^a^	65.18 ± 2.32 ^a^	76.18 ± 1.29 ^a^	50.65 ± 1.81 ^b^	58.09 ± 1.45 ^b^
	Oleacein	3.63 ± 0.27 ^a^	85.76 ± 2.03 ^a^	3.63 ± 0.14 ^a^	85.37 ± 2.81 ^a^	3.17 ± 0.17 ^b^	78.79 ± 1.51 ^b^
	Ligstroside	0.68 ± 0.05 ^a^	69.62 ± 4.25 ^a^	0.75 ± 0.09 ^a^	75.70 ± 3.06 ^a^	0.71 ± 0.17 ^a^	55.40 ± 3.30 ^b^
	Oleuropein aglycone	0.18 ± 0.02 ^a^	80.62 ± 6.25 ^a^	0.19 ± 0.05 ^a^	73.47 ± 3.04 ^a^	0.16 ± 0.02 ^a^	79.87 ± 6.21 ^a^
Pentacyclic triterpenes	Oleanolic acid	13.96 ± 0.05 ^c^	32.60 ± 6.30 ^b^	16.66 ± 0.98 ^b^	38.91 ± 8.22 ^b^	42.79 ± 1.10 ^a^	92.93 ± 4.27 ^a^
	Maslinic acid	0.06 ± 0.01 ^b^	32.07 ± 0.18 ^b^	0.07 ± 0.01 ^b^	22.88 ± 0.27 ^c^	3.06 ± 0.10 ^a^	49.26 ± 0.11 ^a^
Flavonoids and flavonoid glycosides	Luteolin 7-O-glucoside	6.90 ± 0.62 ^a^	88.25 ± 6.77 ^a^	6.32 ± 0.20 ^a^	89.79 ± 1.62 ^a^	5.47 ± 0.23 ^b^	70.65 ± 3.39 ^b^
	Apigenin 7-O-glucoside	3.72 ± 0.36 ^a^	94.38 ± 7.19 ^a^	3.50 ± 0.14 ^a^	93.36 ± 4.22 ^a^	3.55 ± 0.17 ^a^	80.69 ± 4.44 ^b^
	Quercetin	0.43 ± 0.03 ^a^	86.40 ± 4.96 ^a^	0.33 ± 0.02 ^b^	74.33 ± 2.60 ^b^	0.42 ± 0.05 ^a^	86.53 ± 4.15 ^a^
	Quercitrin	0.15 ± 0.01 ^a^	80.62 ± 4.63 ^a^	0.12 ± 0.02 ^a^	73.47 ± 4.69 ^a^	0.14 ± 0.01 ^a^	79.87 ± 4.84 ^a^
Simple phenols	Hydroxy tyrosol	0.97 ± 0.07 ^a^	45.14 ± 4.08 ^a^	0.87 ± 0.08 ^a^	45.68 ± 4.01 ^a^	0.85 ± 0.06 ^a^	52.92 ± 3.78 ^a^
	Chlorogenic acid	0.02 ± 0.01	22.22 ± 2.00	n.d.	0.00	n.d.	0.00

* The results are presented as an average value and standard deviation of three repeated measurements. EE—encapsulation efficiency, AL—liposomes prepared using phospholipids from producer Avanti, PG90—liposomes prepared using granulated phospholipids from producer Lipoid, and PH90—liposomes prepared using hydrogenated phospholipids from producer Lipoid, n.d.—not detected, d.e.—dry extract. The same letter in each row refers to the absence of statistically significant differences (for each variable, i.e., concentration and EE, separately) regarding the results of statistical analysis in one-way analysis of variance and Duncan’s post hoc test at *p* > 0.05 (n = 3).

**Table 3:** Rheological properties of plain and olive leaf extract-loaded liposomes, examined on the 1st and 60th days of storage in the refrigerator.

Day	Liposomes	Viscosity (mPa·s)	Surface Tension (mN/m)	Density (g/cm^3^)
1st	Plain AL	2.63 ± 0.04 ^h,^*	26.4 ± 1.0 ^b^	0.999 ± 0.001 ^c^
	Plain PG90	2.68 ± 0.05 ^h^	15.6 ± 1.3 ^d^	0.999 ± 0.002 ^c^
	Plain PH90	3.07 ± 0.08 ^g^	18.3 ± 0.9 ^c^	1.002 ± 0.003 ^bc^
	AL with extract	6.04 ± 0.03 ^c^	29.6 ± 2.0 ^b^	1.007 ± 0.002 ^ab^
	PG90 with extract	6.78 ± 0.05 ^b^	27.2 ± 0.7 ^b^	1.018 ± 0.011 ^a^
	PH90 with extract	4.49 ± 0.12 ^e^	37.3 ± 1.8 ^a^	1.005 ± 0.002 ^ab^
60th	Plain AL	2.64 ± 0.05 ^h^	17.9 ± 2.4 ^cd^	1.001 ± 0.03 ^bc^
	Plain PG90	3.05 ± 0.11 ^g^	16.0 ± 1.2 ^d^	1.000 ± 0.02 ^bc^
	Plain PH90	3.57 ± 0.06 ^f^	20.3 ± 1.3 ^c^	1.000 ± 0.03 ^bc^
	AL with extract	7.70 ± 0.07 ^a^	28.8 ± 1.5 ^b^	1.008 ± 0.01 ^a^
	PG90 with extract	5.44 ± 0.07 ^d^	28.1 ± 1.0 ^b^	1.011 ± 0.02 ^a^
	PH90 with extract	4.36 ± 0.11 ^e^	39.4 ± 1.9 ^a^	1.004 ± 0.02 ^b^

* The identical letter within each column indicates no statistically significant difference for the respective variable, based on one-way analysis of variance followed by Duncan’s post hoc test at *p* > 0.05 (n = 3). AL—liposomes prepared using phospholipids from producer Avanti, PG90—liposomes prepared using granulated phospholipids from producer Lipoid, PH90—liposomes prepared using hydrogenated phospholipids from producer Lipoid.

## Data Availability

The datasets generated during and/or analyzed during the current study are available from the corresponding author upon reasonable request.

## Supplementary Materials

The following supporting information can be downloaded at https://www.mdpi.com/article/10.3390/ph18111639/s1, Table S1: Results of analytical method validation. Figure S1: HPLC-DAD chromatograms of olive leaf extract with detection at 280 nm (blue line), 250 nm (red line), 330 nm (green line), and 350 nm (purple line). Identified compounds: 1—Hydroxytyrosol, 2—Chlorogenic acid, 3—Luteolin-7-O-glucoside, 4—Quercitrin, 5—Apigenin-7-O-glucoside, 6—Oleuropein, 7—Oleacein, 8—Ligstroside, 9—Quercetin, 10—Oleuropein aglycone. Figure S2: HPLC-DAD chromatograms of encapsulated extract liposome fractions with detection at 250 nm. Identified compounds: 1—Hydroxytyrosol, 2—Chlorogenic acid, 3—Luteolin-7-O-glucoside, 4—Quercitrin, 5—Apigenin-7-O-glucoside, 6—Oleuropein, 7—Oleacein, 8—Ligstroside, 9—Quercetin, 10—Oleuropein aglycone. AL—liposomes prepared using phospholipids from producer Avanti (red line), PG90—liposomes prepared using granulated phospholipids from producer Lipoid (green line), and PH90—liposomes prepared using hydrogenated phospholipids from producer Lipoid (blue line). Table S2: Quantitative analysis of olive leaf extract and extract-loaded liposomes after the 60th day in the refrigerator. Table S3: Quantitative analysis of encapsulated extract fraction in liposomes and encapsulation efficiency after the 60th day in the refrigerator. Figure S3: Structural formulas of the main compounds in olive leaf extract: Oleuropein (Methyl (2S,3E,4S)-4-{2-[2-(3,4-dihydroxyphenyl)ethoxy]-2-oxoethyl}-3-ethylidene-2-{[(2S,3R,4S,5S,6R)-3,4,5-trihydroxy-6-(hydroxymethyl)oxan-2-yl]oxy}-2H-pyran-5-carboxylate); Ligstroside (Methyl (4S,5E,6S)-5-ethylidene-4-[2-[2-(4-hydroxyphenyl)ethoxy]-2-oxoethyl]-6-{[(2S,3R,4S,5S,6R)-3,4,5-trihydroxy-6-(hydroxymethyl)oxan-2-yl]oxy}-4H-pyran-3-carboxylate); Oleacein (2-(3,4-dihydroxyphenyl)ethyl (4Z)-4-formyl-3-(2-oxoethyl)hex-4-enoate); oleuropein-aglycone (Methyl (4S,5E,6R)-4-[2-[2-(3,4-dihydroxyphenyl)ethoxy]-2-oxoethyl]-5-ethylidene-6-hydroxy-4H-pyran-3-carboxylate); Luteolin-7-O-glucoside (2-(3,4-dihydroxyphenyl)-5-hydroxy-7-{[(2S,3R,4S,5S,6R)-3,4,5-trihydroxy-6-(hydroxymethyl)oxan-2-yl]oxy}-4H-chromen-4-one); Apigenin-7-O-glucoside (5-hydroxy-2-(4-hydroxyphenyl)-7-{[(2S,3R,4S,5S,6R)-3,4,5-trihydroxy-6-(hydroxymethyl)oxan-2-yl]oxy}-4H-chromen-4-one). Figure S4. FT-IR spectra of plain liposomes and olive leaf extract; AL—liposomes prepared using phospholipids from producer Avanti, PG90—liposomes prepared using granulated phospholipids from producer Lipoid, PH90—liposomes prepared using hydrogenated phospholipids from producer Lipoid. Figure S5. Antioxidant potential of plain liposomes; values with the same letter for each assay separately and the same number in each sample separately showed no statistically significant difference (*p* > 0.05; n = 3; analysis of variance, Duncan’s *post hoc* test); AL—liposomes prepared using phospholipids from producer Avanti, PG90—liposomes prepared using granulated phospholipids from producer Lipoid, and PH90—liposomes prepared using hydrogenated phospholipids from producer Lipoid. Figure S6: Electrophoretic mobility of plain and olive leaf extract-loaded liposomes over 60 days at 4 °C; AL—liposomes prepared with phospholipids from Avanti; PG90—liposomes with granulated phospholipids (Lipoid); PH90—liposomes with hydrogenated phospholipids (Lipoid). Data represent mean ± standard deviation of triplicate measurements.

## References

[1] Rugini E., De Pace C., Gutiérrez-Pesce P., Muleo R. (2011). Olea. Wild Crop Relatives: Genomic and Breeding Resources.

[2] Hashmi M.A., Khan A., Hanif M., Farooq U., Perveen S. (2015). Traditional Uses, Phytochemistry, and Pharmacology of *Olea europaea* (Olive). Evid. Based Complement. Altern. Med..

[3] Bhattacharjee S., Banerjee A., Anupam D.T. (2024). Role of Ethnomedicinal Resources to Cure Metabolic Diseases. Traditional Resources and Tools for Modern Drug Discovery.

[4] Alesci A., Miller A., Tardugno R., Pergolizzi S. (2022). Chemical Analysis, Biological and Therapeutic Activities of *Olea europaea* L. Extracts. Nat. Prod. Res..

[5] Tsimidou M.Z., Papoti V.T. (2010). Bioactive Ingredients in Olive Leaves. Olives and Olive Oil in Health and Disease Prevention.

[6] Espeso J., Isaza A., Lee J.Y., Sörensen P.M., Jurado P., Avena-Bustillos R.d.J., Olaizola M., Arboleya J.C. (2021). Olive Leaf Waste Management. Front. Sustain. Food Syst..

[7] Selim S., Albqmi M., Al-Sanea M.M., Alnusaire T.S., Almuhayawi M.S., AbdElgawad H., Al Jaouni S.K., Elkelish A., Hussein S., Warrad M. (2022). Valorizing the Usage of Olive Leaves, Bioactive Compounds, Biological Activities, and Food Applications: A Comprehensive Review. Front. Nutr..

[8] Migone C., Cerri L., Ferreira B., Fabiano A., Parri S., Mezzetta A., Guazzelli L., Piras A.M., Zambito Y., Sarmento B. (2025). Olive Leaf Extract-Based Eye Drop Formulations for Corneal Wound Healing. J. Drug Deliv. Sci. Technol..

[9] Panou A.A., Karabagias I.K. (2025). Olive Leaf Extracts as a Medicinal Beverage: Origin, Physico-Chemical Properties, and Bio-Functionality. Beverages.

[10] Aggul A.G., Gulaboglu M., Cetin M., Ozakar E., Ozakar R.S., Aydin T. (2020). Effects of Emulsion Formulations of Oleuropein Isolated from Ethanol Extract of Olive Leaf in Diabetic Rats. An. Acad. Bras. Cienc..

[11] Ranalli A., Contento S., Lucera L., Di Febo M., Marchegiani D., Di Fonzo V. (2006). Factors Affecting the Contents of Iridoid Oleuropein in Olive Leaves (*Olea europaea* L.). J. Agric. Food Chem..

[12] Benavente-García O., Castillo J., Lorente J., Ortuño A., Del Rio J. (2000). Antioxidant Activity of Phenolics Extracted from *Olea europaea* L. Leaves. Food Chem..

[13] Ahamad J., Toufeeq I., Khan M.A., Ameen M.S.M., Anwer E.T., Uthirapathy S., Mir S.R., Ahmad J. (2019). Oleuropein: A Natural Antioxidant Molecule in the Treatment of Metabolic Syndrome. Phyther. Res..

[14] Qabaha K., AL-Rimawi F., Qasem A., Naser S.A. (2018). Oleuropein Is Responsible for the Major Anti-Inflammatory Effects of Olive Leaf Extract. J. Med. Food.

[15] Ahmadvand H., Noori A., Dehnoo M.G., Bagheri S., Cheraghi R.A. (2014). Hypoglycemic, Hypolipidemic and Antiatherogenic Effects of Oleuropein in Alloxan-Induced Type 1 Diabetic Rats. Asian Pacific J. Trop. Dis..

[16] Topuz S., Bayram M. (2022). Oleuropein Extraction from Leaves of Three Olive Varieties (*Olea europaea* L.): Antioxidant and Antimicrobial Properties of Purified Oleuropein and Oleuropein Extracts. J. Food Process. Preserv..

[17] Kabbash E.M., Abdel-Shakour Z.T., El-Ahmady S.H., Wink M., Ayoub I.M. (2023). Comparative Metabolic Profiling of Olive Leaf Extracts from Twelve Different Cultivars Collected in Both Fruiting and Flowering Seasons. Sci. Rep..

[18] Dzubak P., Hajduch M., Vydra D., Hustova A., Kvasnica M., Biedermann D., Markova L., Urban M., Sarek J. (2006). Pharmacological Activities of Natural Triterpenoids and Their Therapeutic Implications. Nat. Prod. Rep..

[19] Kuo R.-Y., Qian K., Morris-Natschke S.L., Lee K.-H. (2009). Plant-Derived Triterpenoids and Analogues as Antitumor and Anti-HIV Agents. Nat. Prod. Rep..

[20] Castellano J.M., Ramos-Romero S., Perona J.S. (2022). Oleanolic Acid: Extraction, Characterization and Biological Activity. Nutrients.

[21] Nikou T., Sakavitsi M.E., Kalampokis E., Halabalaki M. (2022). Metabolism and Bioavailability of Olive Bioactive Constituents Based on In Vitro, In Vivo and Human Studies. Nutrients.

[22] Žugčić T., Abdelkebir R., Alcantara C., Collado M.C., García-Pérez J.V., Meléndez-Martínez A.J., Režek Jambrak A., Lorenzo J.M., Barba F.J. (2019). From Extraction of Valuable Compounds to Health Promoting Benefits of Olive Leaves through Bioaccessibility, Bioavailability and Impact on Gut Microbiota. Trends Food Sci. Technol..

[23] Batarfi W.A., Yunus M.H.M., Hamid A.A., Lee Y.T., Maarof M. (2024). Hydroxytyrosol: A Promising Therapeutic Agent for Mitigating Inflammation and Apoptosis. Pharmaceutics.

[24] Galmés S., Reynés B., Palou M., Palou-March A., Palou A. (2021). Absorption, Distribution, Metabolism, and Excretion of the Main Olive Tree Phenols and Polyphenols: A Literature Review. J. Agric. Food Chem..

[25] Bergonzi M.C., De Stefani C., Vasarri M., Ivanova Stojcheva E., Ramos-Pineda A.M., Baldi F., Bilia A.R., Degl’Innocenti D. (2023). Encapsulation of Olive Leaf Polyphenol-Rich Extract in Polymeric Micelles to Improve Its Intestinal Permeability. Nanomaterials.

[26] Di Lorenzo C., Colombo F., Biella S., Stockley C., Restani P. (2021). Polyphenols and Human Health: The Role of Bioavailability. Nutrients.

[27] Qiao K., Zhao M., Huang Y., Liang L., Zhang Y. (2024). Bitter Perception and Effects of Foods Rich in Bitter Compounds on Human Health: A Comprehensive Review. Foods.

[28] Jovanović A.A., Balanč B., Petrović P.M., Volić M., Micić D., Živković J., Šavikin K.P. (2024). Design and Characterization of Liposomal-Based Carriers for the Encapsulation of *Rosa canina* Fruit Extract: In Vitro Gastrointestinal Release Behavior. Plants.

[29] Dinić S., Arambašić Jovanović J., Uskoković A., Jovanović A., Grdović N., Rajić J., Đorđević M., Sarić A., Bugarski B., Vidaković M. (2024). Liposome Encapsulation Enhances the Antidiabetic Efficacy of Silibinin. Pharmaceutics.

[30] Lee M.-K. (2020). Liposomes for Enhanced Bioavailability of Water-Insoluble Drugs: In Vivo Evidence and Recent Approaches. Pharmaceutics.

[31] Nsairat H., Khater D., Sayed U., Odeh F., Al Bawab A., Alshaer W. (2022). Liposomes: Structure, Composition, Types, and Clinical Applications. Heliyon.

[32] Kashapov R., Ibragimova A., Pavlov R., Gabdrakhmanov D., Kashapova N., Burilova E., Zakharova L., Sinyashin O. (2021). Nanocarriers for Biomedicine: From Lipid Formulations to Inorganic and Hybrid Nanoparticles. Int. J. Mol. Sci..

[33] Paramshetti S., Angolkar M., Talath S., Osmani R.A.M., Spandana A., Al Fatease A., Hani U., Ramesh K.V.R.N.S., Singh E. (2024). Unravelling the in Vivo Dynamics of Liposomes: Insights into Biodistribution and Cellular Membrane Interactions. Life Sci..

[34] Jovanović A.A., Balanč B., Volić M., Pećinar I., Živković J., Šavikin K.P. (2023). Rosehip Extract-Loaded Liposomes for Potential Skin Application: Physicochemical Properties of Non- and UV-Irradiated Liposomes. Plants.

[35] Feng Y., Sun C., Yuan Y., Zhu Y., Wan J., Firempong C.K., Omari-Siaw E., Xu Y., Pu Z., Yu J. (2016). Enhanced Oral Bioavailability and in Vivo Antioxidant Activity of Chlorogenic Acid via Liposomal Formulation. Int. J. Pharm..

[36] Tatipamula V.B., Kukavica B. (2021). Phenolic Compounds as Antidiabetic, Anti-inflammatory, and Anticancer Agents and Improvement of Their Bioavailability by Liposomes. Cell Biochem. Funct..

[37] Faridi Esfanjani A., Assadpour E., Jafari S.M. (2018). Improving the Bioavailability of Phenolic Compounds by Loading Them within Lipid-Based Nanocarriers. Trends Food Sci. Technol..

[38] Lombardo D., Kiselev M.A. (2022). Methods of Liposomes Preparation: Formation and Control Factors of Versatile Nanocarriers for Biomedical and Nanomedicine Application. Pharmaceutics.

[39] Camilleri D., Attard K., Lia F. (2025). Formulation and Evaluation of Liposome-Encapsulated Phenolic Compounds from Olive Mill Waste: Insights into Encapsulation Efficiency, Antioxidant, and Cytotoxic Activities. Molecules.

[40] Bonechi C., Donati A., Tamasi G., Pardini A., Rostom H., Leone G., Lamponi S., Consumi M., Magnani A., Rossi C. (2019). Chemical Characterization of Liposomes Containing Nutraceutical Compounds: Tyrosol, Hydroxytyrosol and Oleuropein. Biophys. Chem..

[41] Prevete G., Donati E., Ruggiero A.P., Fardellotti S., Lilla L., Ramundi V., Nicoletti I., Mariani F., Mazzonna M. (2024). Encapsulation of *Olea europaea* Leaf Polyphenols in Liposomes: A Study on Their Antimicrobial Activity to Turn a Byproduct into a Tool to Treat Bacterial Infection. ACS Appl. Mater. Interfaces.

[42] Tavakoli H., Hosseini O., Jafari S.M., Katouzian I. (2018). Evaluation of Physicochemical and Antioxidant Properties of Yogurt Enriched by Olive Leaf Phenolics within Nanoliposomes. J. Agric. Food Chem..

[43] Talhaoui N., Taamalli A., Gómez-Caravaca A.M., Fernández-Gutiérrez A., Segura-Carretero A. (2015). Phenolic Compounds in Olive Leaves: Analytical Determination, Biotic and Abiotic Influence, and Health Benefits. Food Res. Int..

[44] Ghasemi S., Koohi D.E., Emmamzadehhashemi M.S.B., Khamas S.S., Moazen M., Hashemi A.K., Amin G., Golfakhrabadi F., Yousefi Z., Yousefbeyk F. (2018). Investigation of Phenolic Compounds and Antioxidant Activity of Leaves Extracts from Seventeen Cultivars of Iranian Olive (*Olea europaea* L.). J. Food Sci. Technol..

[45] Aouidi F., Dupuy N., Artaud J., Roussos S., Msallem M., Perraud Gaime I., Hamdi M. (2012). Rapid Quantitative Determination of Oleuropein in Olive Leaves (*Olea europaea*) Using Mid-Infrared Spectroscopy Combined with Chemometric Analyses. Ind. Crops Prod..

[46] Tarchi I., Olewnik-Kruszkowska E., Aït-Kaddour A., Bouaziz M. (2025). Innovative Process for the Recovery of Oleuropein-Rich Extract from Olive Leaves and Its Biological Activities: Encapsulation for Activity Preservation with Concentration Assessment Pre and Post Encapsulation. ACS Omega.

[47] Cristiano M.C., Froiio F., Mancuso A., Cosco D., Dini L., Di Marzio L., Fresta M., Paolino D. (2021). Oleuropein-Laded Ufasomes Improve the Nutraceutical Efficacy. Nanomaterials.

[48] González-Ortega R., Šturm L., Skrt M., Di Mattia C.D., Pittia P., Poklar Ulrih N. (2021). Liposomal Encapsulation of Oleuropein and an Olive Leaf Extract: Molecular Interactions, Antioxidant Effects and Applications in Model Food Systems. Food Biophys..

[49] Nassir A.M., Ibrahim I.A.A., Md S., Waris M., Tanuja, Ain M.R., Ahmad I., Shahzad N. (2019). Surface Functionalized Folate Targeted Oleuropein Nano-Liposomes for Prostate Tumor Targeting: In Vitro and in Vivo Activity. Life Sci..

[50] Portaccio M., Faramarzi B., Lepore M. (2023). Probing Biochemical Differences in Lipid Components of Human Cells by Means of ATR-FTIR Spectroscopy. Biophysica.

[51] Chia N.C., Mendelsohn R. (1996). Conformational disorder in unsaturated phospholipids by FTIR spectroscopy. Biochim. Biophys. Acta..

[52] Kechagias A., Leontiou A.A., Vardakas A., Stathopoulos P., Xenaki M., Stathopoulou P., Proestos C., Giannelis E.P., Chalmpes N., Salmas C.E. (2025). Antioxidant Nanohybrid Materials Derived via Olive Leaf Extract Incorporation in Layered Double Hydroxide: Preparation, Characterization, and Evaluation for Applications. Antioxidants.

[53] Agatonovic-Kustrin S., Gegechkori V., Petrovich D.S., Ilinichna K.T., Morton D.W. (2021). HPTLC and FTIR Fingerprinting of Olive Leaves Extracts and ATR-FTIR Characterisation of Major Flavonoids and Polyphenolics. Molecules.

[54] Scarpi-Luttenauer M., Boubegtiten-Fezoua Z., Hellwig P., Chaumont A., Vincent B., Barloy L., Mobian P. (2025). Spectroscopic Evidence of the Interaction of Titanium(IV) Coordination Complexes with a Phosphate Head Group in Phospholipids. Dalton Trans..

[55] Raederstorff D. (2009). Antioxidant activity of olive polyphenols in humans: A review. Int. J. Vitam. Nutr. Res..

[56] Lins P.G., Pugine S.M.P., Scatolini A.M., de Melo M.P. (2018). In vitro antioxidant activity of olive leaf extract (*Olea europaea* L.) and its protective effect on oxidative damage in human erythrocytes. Heliyon.

[57] Xie P., Huang L., Zhang C., Zhang Y. (2015). Phenolic compositions, and antioxidant performance of olive leaf and fruit (*Olea europaea* L.) extracts and their structure–activity relationships. J. Funct. Foods..

[58] Ilgaz C., Casula L., Sarais G., Schlich M., Dessì D., Cardia M.C., Sinico C., Kadiroglu P., Lai F. (2025). Proniosomal encapsulation of olive leaf extract for improved delivery of oleuropein: Towards the valorization of an agro-industrial byproduct. Food Chem..

[59] Yuan J.-J., Qin F.G.F., Tu J.-L., Li B. (2017). Preparation, Characterization, and Antioxidant Activity Evaluation of Liposomes Containing Water-Soluble Hydroxytyrosol from Olive. Molecules.

[60] García K.P., Zarschler K., Barbaro L., Barreto J.A., O’Malley W., Spiccia L., Stephan H., Graham B. (2014). Zwitterionic-Coated “Stealth” Nanoparticles for Biomedical Applications: Recent Advances in Countering Biomolecular Corona Formation and Uptake by the Mononuclear Phagocyte System. Small.

[61] Zhang L., Pornpattananangkul D., Hu C.-M., Huang C.-M. (2010). Development of Nanoparticles for Antimicrobial Drug Delivery. Curr. Med. Chem..

[62] Roberts S.A., Lee C., Singh S., Agrawal N. (2022). Versatile Encapsulation and Synthesis of Potent Liposomes by Thermal Equilibration. Membranes.

[63] Micheletto Y.M.S., Jesus B.V.d., Peres G.L., Pinto V.Z. (2025). A Systematic Preparation of Liposomes with Yerba Mate (*Ilex paraguariensis*) Extract. Plants.

[64] Yu X., Chu S., Hagerman A.E., Lorigan G.A. (2011). Probing the Interaction of Polyphenols with Lipid Bilayers by Solid-State NMR Spectroscopy. J. Agric. Food Chem..

[65] Uekusa Y., Kamihira M., Nakayama T. (2007). Dynamic Behavior of Tea Catechins Interacting with Lipid Membranes As Determined by NMR Spectroscopy. J. Agric. Food Chem..

[66] Kajiya K., Kumazawa S., Naito A., Nakayama T. (2008). Solid-state NMR Analysis of the Orientation and Dynamics of Epigallocatechin Gallate, a Green Tea Polyphenol, Incorporated into Lipid Bilayers. Magn. Reson. Chem..

[67] Phan H.T.T., Yoda T., Chahal B., Morita M., Takagi M., Vestergaard M.C. (2014). Structure-Dependent Interactions of Polyphenols with a Biomimetic Membrane System. Biochim. Biophys. Acta Biomembr..

[68] Dag D., Oztop M.H. (2017). Formation and Characterization of Green Tea Extract Loaded Liposomes. J. Food Sci..

[69] Aisha A.F., Majid A.M.S.A., Ismail Z. (2014). Preparation and Characterization of Nano Liposomes of *Orthosiphon stamineus* Ethanolic Extract in Soybean Phospholipids. BMC Biotechnol..

[70] Jovanović A.A., Balanč B.D., Djordjević V.B., Ota A., Skrt M., Šavikin K.P., Bugarski B.M., Nedović V.A., Ulrih N.P. (2019). Effect of Gentisic Acid on the Structural-Functional Properties of Liposomes Incorporating β-Sitosterol. Colloid. Surf. B Biointerfaces.

[71] Song F., Yang G., Wang Y., Tian S. (2022). Effect of Phospholipids on Membrane Characteristics and Storage Stability of Liposomes. Innov. Food Sci. Emerg. Technol..

[72] Tai K., Rappolt M., Mao L., Gao Y., Yuan F. (2020). Stability and Release Performance of Curcumin-Loaded Liposomes with Varying Content of Hydrogenated Phospholipids. Food Chem..

[73] Leekumjorn S., Cho H.-J., Wu Y., Wright N.T., Sum A.K., Chan C. (2009). The Role of Fatty Acid Unsaturation in Minimizing Biophysical Changes on the Structure and Local Effects of Bilayer Membranes. Biochim. Biophys. Acta Biomembr..

[74] Amărandi R.-M., Neamṭu A., Ştiufiuc R.-I., Marin L., Drăgoi B. (2024). Impact of Lipid Composition on Vesicle Protein Adsorption: A BSA Case Study. ACS Omega.

[75] Subczynski W., Wisniewska-Becker A. (2000). Physical Properties of Lipid Bilayer Membranes: Relevance to Membrane Biological Functions. Acta Biochim. Pol..

[76] Howard F.B., Levin I.W. (2010). Lipid Vesicle Aggregation Induced by Cooling. Int. J. Mol. Sci..

[77] Danaei M., Dehghankhold M., Ataei S., Hasanzadeh Davarani F., Javanmard R., Dokhani A., Khorasani S., Mozafari M.R. (2018). Impact of Particle Size and Polydispersity Index on the Clinical Applications of Lipidic Nanocarrier Systems. Pharmaceutics.

[78] Samad A., Sultana Y., Aqil M. (2007). Liposomal Drug Delivery Systems: An Update Review. Curr. Drug Deliv..

[79] Shashidhar G.M., Manohar B. (2018). Nanocharacterization of Liposomes for the Encapsulation of Water Soluble Compounds from *Cordyceps sinensis* CS1197 by a Supercritical Gas Anti-Solvent Technique. RSC Adv..

[80] Narenji M., Talaee M.R., Moghimi H.R. (2016). Investigating the Effects of Size, Charge, Viscosity and Bilayer Flexibility on Liposomal Delivery under Convective Flow. Int. J. Pharm..

[81] Jovanović A.A., Balanč B.D., Ota A., Ahlin Grabnar P., Djordjević V.B., Šavikin K.P., Bugarski B.M., Nedović V.A., Poklar Ulrih N. (2018). Comparative Effects of Cholesterol and Β-Sitosterol on the Liposome Membrane Characteristics. Eur. J. Lipid Sci. Technol..

[82] Salarbashi D., Tafaghodi M., Fathi M., Aboutorabzade S.M., Sabbagh F. (2021). Development of Curcumin-loaded *Prunus armeniaca* Gum Nanoparticles: Synthesis, Characterization, Control Release Behavior, and Evaluation of Anticancer and Antimicrobial Properties. Food Sci. Nutr..

[83] Prevete G., Carvalho L.G., del Carmen Razola-Diaz M., Verardo V., Mancini G., Fiore A., Mazzonna M. (2024). Ultrasound Assisted Extraction and Liposome Encapsulation of Olive Leaves and Orange Peels: How to Transform Biomass Waste into Valuable Resources with Antimicrobial Activity. Ultrason. Sonochem..

[84] Moraes F.C., Pichon C., Letourneur D., Chaubet F. (2021). MiRNA Delivery by Nanosystems: State of the Art and Perspectives. Pharmaceutics.

[85] Olfati A., Karimi N., Arkan E., Zhaleh M., Mozafari M.R. (2025). Enhancing Bioavailability and Stability of Plant Secondary Metabolites: Formulation and Characterization of Nanophytosomes Encapsulating Red Bryony and Horned Poppy Extracts. J. Funct. Biomater..

[86] Guldiken B., Gibis M., Boyacioglu D., Capanoglu E., Weiss J. (2018). Physical and Chemical Stability of Anthocyanin-Rich Black Carrot Extract-Loaded Liposomes During Storage. Food Res. Int..

[87] Toopkanloo S.P., Tan T.B., Abas F., Alharthi F.A., Nehdi I.A., Tan C.P. (2020). Impact of Quercetin Encapsulation with Added Phytosterols on Bilayer Membrane and Photothermal-Alteration of Novel Mixed Soy Lecithin-Based Liposome. Nanomaterials.

[88] Baranauskaite J., Duman G., Corapcıoğlu G., Baranauskas A., Taralp A., Ivanauskas L., Bernatoniene J. (2018). Liposomal Incorporation to Improve Dissolution and Stability of Rosmarinic Acid and Carvacrol Extracted from Oregano (*O. onites* L.). Biomed Res. Int..

[89] Ruozi B., Tosi G., Forni F., Fresta M., Vandelli M.A. (2005). Atomic Force Microscopy and Photon Correlation Spectroscopy: Two Techniques for Rapid Characterization of Liposomes. Eur. J. Pharm. Sci..

[90] Leitgeb M., Knez Ž., Primožič M. (2020). Sustainable Technologies for Liposome Preparation. J. Supercrit. Fluids.

[91] Bompard J., Rosso A., Brizuela L., Mebarek S., Blum L.J., Trunfio-Sfarghiu A.M., Lollo G., Granjon T., Girard-Egrot A., Maniti O. (2020). Membrane Fluidity as a New Means to Selectively Target Cancer Cells with Fusogenic Lipid Carriers. Langmuir.

[92] Kaddah S., Khreich N., Kaddah F., Charcosset C., Greige-Gerges H. (2018). Cholesterol Modulates the Liposome Membrane Fluidity and Permeability for a Hydrophilic Molecule. Food Chem. Toxicol..

[93] Duffy C.F., Gafoor S., Richards D.P., Admadzadeh H., O’Kennedy R., Arriaga E.A. (2001). Determination of Properties of Individual Liposomes by Capillary Electrophoresis with Postcolumn Laser-Induced Fluorescence Detection. Anal. Chem..

[94] Yu M., Song W., Tian F., Dai Z., Zhu Q., Ahmad E., Guo S., Zhu C., Zhong H., Yuan Y. (2019). Temperature- and Rigidity-Mediated Rapid Transport of Lipid Nanovesicles in Hydrogels. Proc. Natl. Acad. Sci. USA.

[95] Antohe F., Lin L., Kao G.Y., Poznansky M.J., Allen T.M. (2004). Transendothelial Movement of Liposomes In Vitro Mediated by Cancer Cells, Neutrophils or Histamine. J. Liposome Res..

[96] Qian S., Li C., Zuo Z. (2012). Pharmacokinetics and Disposition of Various Drug Loaded Liposomes. Curr. Drug Metab..

[97] Abreu A.S., Castanheira E.M., Queiroz M.-J.R., Ferreira P.M., Vale-Silva L.A., Pinto E. (2011). Nanoliposomes for Encapsulation and Delivery of the Potential Antitumoral Methyl 6-Methoxy-3-(4-Methoxyphenyl)-1H-Indole-2-Carboxylate. Nanoscale Res. Lett..

[98] Immordino M.L., Dosio F., Cattel L. (2006). Stealth Liposomes: Review of the Basic Science, Rationale, and Clinical Applications, Existing and Potential. Int. J. Nanomed..

[99] Schlieper P., Medda P.K., Kaufmann R. (1981). Drug-Induced Zeta Potential Changes in Liposomes Studied by Laser Doppler Spectroscopy. Biochim. Biophys. Acta Biomembr..

[100] Yang D., Wang X.-Y., Gan L.-J., Zhang H., Shin J.-A., Lee K.-T., Hong S.-T. (2015). Effects of Flavonoid Glycosides Obtained from a *Ginkgo biloba* Extract Fraction on the Physical and Oxidative Stabilities of Oil-in-Water Emulsions Prepared from a Stripped Structured Lipid with a Low Omega-6 to Omega-3 Ratio. Food Chem..

[101] Rudzińska M., Grygier A., Knight G., Kmiecik D. (2024). Liposomes as Carriers of Bioactive Compounds in Human Nutrition. Foods.

[102] Chen Y., Arriaga E.A. (2007). Individual Electrophoretic Mobilities of Liposomes and Acidic Organelles Displaying PH Gradients Across Their Membranes. Langmuir.

[103] Giannopoulos-Dimitriou A., Saiti A., Petrou A., Vizirianakis I.S., Fatouros D.G. (2024). Liposome Stability and Integrity. Liposomes in Drug Delivery.

[104] Pattni B.S., Chupin V.V., Torchilin V.P. (2015). New Developments in Liposomal Drug Delivery. Chem. Rev..

[105] Figueroa-Robles A., Antunes-Ricardo M., Guajardo-Flores D. (2021). Encapsulation of Phenolic Compounds with Liposomal Improvement in the Cosmetic Industry. Int. J. Pharm..

[106] Savaghebi D., Barzegar M., Mozafari M.R. (2020). Manufacturing of Nanoliposomal Extract from *Sargassum boveanum* Algae and Investigating Its Release Behavior and Antioxidant Activity. Food Sci. Nutr..

[107] de Assis L.M., Machado A.R., de Souza da Motta A., Costa J.A.V., de Souza-Soares L.A. (2014). Development and Characterization of Nanovesicles Containing Phenolic Compounds of Microalgae *Spirulina* Strain LEB-18 and *Chlorella pyrenoidosa*. Adv. Mater. Phys. Chem..

[108] Yarce C.J., Alhajj M.J., Sanchez J.D., Oñate-Garzón J., Salamanca C.H. (2020). Development of Antioxidant-Loaded Nanoliposomes Employing Lecithins with Different Purity Grades. Molecules.

[109] Yuda T., Maruyama K., Takizawa T., Iwatsuru M. (1994). Long-Circulating Liposomes. Drug Deliv. Syst..

[110] Ishida T., Harashima H., Kiwada H. (2001). Interactions of Liposomes with Cells In Vitro and In Vivo: Opsonins and Receptors. Curr. Drug Metab..

[111] Qi X.-R., Zhao Z. (2011). Comparative Study of the in Vitro and in Vivo Characteristics of Cationic and Neutral Liposomes. Int. J. Nanomed..

[112] Sharifiaghdam M., Sun X., Leung A.W.Y., Dos Santos N., Wretham N., Nosrati Z., Bally M.B. (2025). Liposomal Formulations of Metal-CX5461 Complexes: Copper-CX5461 Complexation Mediates CX5461 Degradation while Zinc-CX5461 Formulations Are Suitable for Development. J. Drug Deliv. Sci. Technol..

[113] Ramsay E., Alnajim J., Anantha M., Taggar A., Thomas A., Edwards K., Karlsson G., Webb M., Bally M. (2006). Transition Metal-Mediated Liposomal Encapsulation of Irinotecan (CPT-11) Stabilizes the Drug in the Therapeutically Active Lactone Conformation. Pharm. Res..

[114] Demir B., Barlas F.B., Guler E., Gumus P.Z., Can M., Yavuz M., Coskunol H., Timur S. (2014). Gold Nanoparticle Loaded Phytosomal Systems: Synthesis, Characterization and in Vitro Investigations. RSC Adv..

[115] Safta D.A., Bogdan C., Moldovan M.L. (2022). Vesicular Nanocarriers for Phytocompounds in Wound Care: Preparation and Characterization. Pharmaceutics.

[116] Hammoud Z., Kayouka M., Trifan A., Sieniawska E., Jemâa J.M.B., Elaissari A., Greige-Gerges H. (2021). Encapsulation of α-Pinene in Delivery Systems Based on Liposomes and Cyclodextrins. Molecules.

[117] Fernandes F., Dias-Teixeira M., Delerue-Matos C., Grosso C. (2021). Critical Review of Lipid-Based Nanoparticles as Carriers of Neuroprotective Drugs and Extracts. Nanomaterials.

[118] Wahyudiono, He J., Hu X., Machmudah S., Yasuda K., Takami S., Kanda H., Goto M. (2022). Curcumin-Loaded Liposome Preparation in Ultrasound Environment under Pressurized Carbon Dioxide. Foods.

[119] Hac-Wydro K., Wydro P. (2007). The Influence of Fatty Acids on Model Cholesterol/Phospholipid Membranes. Chem. Phys. Lipids.

[120] Fitzgerald J.E., Venable R.M., Pastor R.W., Lyman E.R. (2023). Surface Viscosities of Lipid Bilayers Determined from Equilibrium Molecular Dynamics Simulations. Biophys. J..

[121] Jara-Quijada E., Pérez-Won M., Tabilo-Munizaga G., Lemus-Mondaca R., González-Cavieres L., Palma-Acevedo A., Herrera-Lavados C. (2024). Liposomes Loaded with Green Tea Polyphenols—Optimization, Characterization, and Release Kinetics Under Conventional Heating and Pulsed Electric Fields. Food Bioprocess Technol..

[122] Karaz S., Senses E. (2023). Liposomes Under Shear: Structure, Dynamics, and Drug Delivery Applications. Adv. NanoBiomed Res..

[123] Santos S., Neves A.R., Silva A., Barbosa M., Reis S., Barbosa J. (2016). Nanostructured Lipid Carriers Loaded with Resveratrol Modulate Human Dendritic Cells. Int. J. Nanomed..

[124] Budai L., Budai M., Fülöpné Pápay Z.E., Szalkai P., Niczinger N.A., Kijima S., Sugibayashi K., Antal I., Kállai-Szabó N. (2023). Viscoelasticity of Liposomal Dispersions. Nanomaterials.

[125] Ghazvini S., Alonso R., Alhakamy N., Dhar P. (2018). pH-Induced Changes in the Surface Viscosity of Unsaturated Phospholipids Monitored Using Active Interfacial Microrheology. Langmuir.

[126] Zeng W., Li P., Huang Y., Xia A., Zhu X., Zhu X., Liao Q. (2022). How Interfacial Properties Affect Adhesion: An Analysis from the Interactions between Microalgal Cells and Solid Substrates. Langmuir.

[127] Zaborowska M., Dobrowolski M.A., Matyszewska D. (2023). Revealing the Structure and Mechanisms of Action of a Synthetic Opioid with Model Biological Membranes at the Air-Water Interface. Colloid. Surf. B Biointerfaces.

[128] Yasui K., Tuziuti T., Kanematsu W. (2023). Mechanism of the Decrease in Surface Tension by Bulk Nanobubbles (Ultrafine Bubbles). Langmuir.

[129] Söderlund T., Alakoskela J.-M.I., Pakkanen A.L., Kinnunen P.K.J. (2003). Comparison of the Effects of Surface Tension and Osmotic Pressure on the Interfacial Hydration of a Fluid Phospholipid Bilayer. Biophys. J..

[130] Malekar S.A., Sarode A.L., Bach A.C., Worthen D.R. (2016). The Localization of Phenolic Compounds in Liposomal Bilayers and Their Effects on Surface Characteristics and Colloidal Stability. AAPS PharmSciTech..

[131] Karonen M. (2022). Insights into Polyphenol–Lipid Interactions: Chemical Methods, Molecular Aspects and Their Effects on Membrane Structures. Plants.

[132] Fathi Azarbayjani A., Jouyban A., Chan S.Y. (2009). Impact of Surface Tension in Pharmaceutical Sciences. J. Pharm. Pharm. Sci..

[133] Păvăloiu R.-D., Sha’at F., Bubueanu C., Deaconu M., Neagu G., Sha’at M., Anastasescu M., Mihailescu M., Matei C., Nechifor G. (2020). Polyphenolic Extract from *Sambucus ebulus* L. Leaves Free and Loaded into Lipid Vesicles. Nanomaterials.

[134] Iversen A., Utterström J., Khare L.P., Aili D. (2024). Influence of Lipid Vesicle Properties on the Function of Conjugation Dependent Membrane Active Peptides. J. Mater. Chem. B.

[135] Kaeswurm J.A.H., Scharinger A., Teipel J., Buchweitz M. (2021). Absorption Coefficients of Phenolic Structures in Different Solvents Routinely Used for Experiments. Molecules.

[136] Pjanović R., Bošković-Vragolović N., Veljković-Giga J., Garić-Grulović R., Pejanović S., Bugarski B. (2010). Diffusion of Drugs from Hydrogels and Liposomes as Drug Carriers. J. Chem. Technol. Biotechnol..

[137] Taira M.C., Chiaramoni N.S., Pecuch K.M., Alonso-Romanowski S. (2004). Stability of Liposomal Formulations in Physiological Conditions for Oral Drug Delivery. Drug Deliv..

[138] Gibis M., Ruedt C., Weiss J. (2016). In Vitro Release of Grape-Seed Polyphenols Encapsulated from Uncoated and Chitosan-Coated Liposomes. Food Res. Int..

[139] Duque-Soto C., Leyva-Jiménez F.J., Quirantes-Piné R., López-Bascón M.A., Lozano-Sánchez J., Borrás-Linares I. (2024). Evaluation of Olive Leaf Phenolic Compounds’ Gastrointestinal Stability Based on Co-Administration and Microencapsulation with Non-Digestible Carbohydrates. Nutrients.

[140] Paulo F., Santos L. (2021). Deriving Valorization of Phenolic Compounds from Olive Oil By-Products for Food Applications through Microencapsulation Approaches: A Comprehensive Review. Crit. Rev. Food Sci. Nutr..

[141] Flamminii F., Di Mattia C.D., Nardella M., Chiarini M., Valbonetti L., Neri L., Difonzo G., Pittia P. (2020). Structuring Alginate Beads with Different Biopolymers for the Development of Functional Ingredients Loaded with Olive Leaves Phenolic Extract. Food Hydrocoll..

[142] Caligiani A., Malavasi G., Palla G., Marseglia A., Tognolini M., Bruni R. (2013). A Simple GC–MS Method for the Screening of Betulinic, Corosolic, Maslinic, Oleanolic and Ursolic Acid Contents in Commercial Botanicals Used as Food Supplement Ingredients. Food Chem..

[143] Mohammadi M., Hamishehkar H., Ghorbani M., Shahvalizadeh R., Pateiro M., Lorenzo J.M. (2021). Engineering of Liposome Structure to Enhance Physicochemical Properties of *Spirulina plantensis* Protein Hydrolysate: Stability during Spray-Drying. Antioxidants.

