# Supplementary material for: Valorization of Olive Leaf Extract via Tailored Liposomal Carriers: Comparative Analysis of Physicochemical Features, Antioxidant Capacity, and Stability
Source: Pharmaceuticals (Basel). 2025 Oct 30;18(11):1639. doi: 10.3390/ph18111639 (PMC12655330; doi:10.3390/ph18111639)
Supplement: Supplementary file 1 [file pharmaceuticals-18-01639-s001.zip › pharmaceuticals-3949166-supplementary.pdf]

Table S1. Results of analytical method validation.

Analyte	Equation	R^2	Precision (RSD (%))		Recovery (%)	LOD (μg/mL)	LOQ (μg/mL)	Linearity range (μg/mL)	U* (%)
			Intra-day	Inter-day					
Oleuropein	y=4.9837x	0.9997	0.55	4.96	93-105	5	10	100-3000	12
Oleacein	y=2.3765x	0.9959	0.31	4.10	95-104	10	20	25-300	10
Ligstroside	y=7.3766x	0.9996	4.87	6.46	94-105	1	5	5-150	18
Oleuropein aglycone	y=8.1201x	0.9934	2.53	4.81	90-104	5	10	10-300	14
Oleanolic acid	y=3415.5x	0.9911	3.23	5.93	91-102	1	5	10-250	11
Maslinic acid	y=2747.3x	0.9936	3.83	5.23	89-101	5	10	10-250	13
Luteolin 7-O-glucoside	y=19.147x	0.9982	2.02	3.58	92-106	0.5	1	4-120	10
Apigenin-7-O-glucoside	y=19.767x	0.9988	1.92	4.74	90-104	1	5	10-300	12
Quercetin	y=20.703x	0.9990	2.42	3.62	93-107	1	5	10-300	11
Quercitrin	y=17.782x	0.9992	1.70	4.33	92-105	5	10	10-300	12
Hydroxytyrosol	y=7.0798x	0.9996	2.69	5.29	92-104	1	5	10-250	11
Chlorogenic acid	y=18.457x	0.9994	2.30	4.20	94-105	0.5	1	10-250	10

*U-expanded measuring uncertainty (k=2); LOD, Limit of Detection; LOQ, Limit of Quantification.

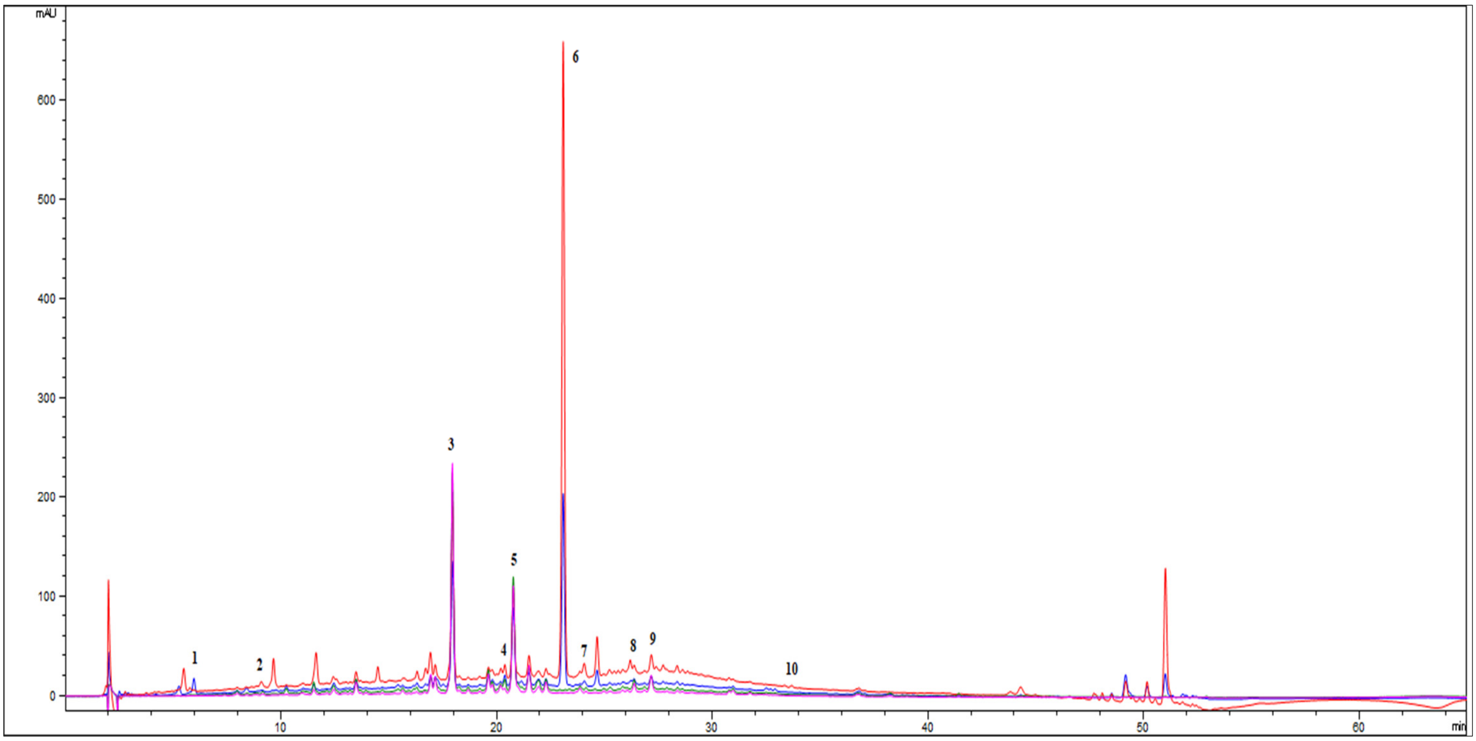

Figure S1. HPLC-DAD chromatograms of olive leaf extract with detection at 280 nm (blue line), 250 nm (red line), 330 nm (green line), and 350 nm (purple line). Identified compounds: 1 - Hydroxytyrosol, 2 - Chlorogenic acid, 3 - Luteolin-7-O-glucoside, 4 - Quercitrin, 5 - Apigenin-7-O-glucoside, 6 - Oleuropein, 7 - Oleacein, 8 - Ligstroside, 9 - Quercetin, 10 - Oleuropein aglycone.

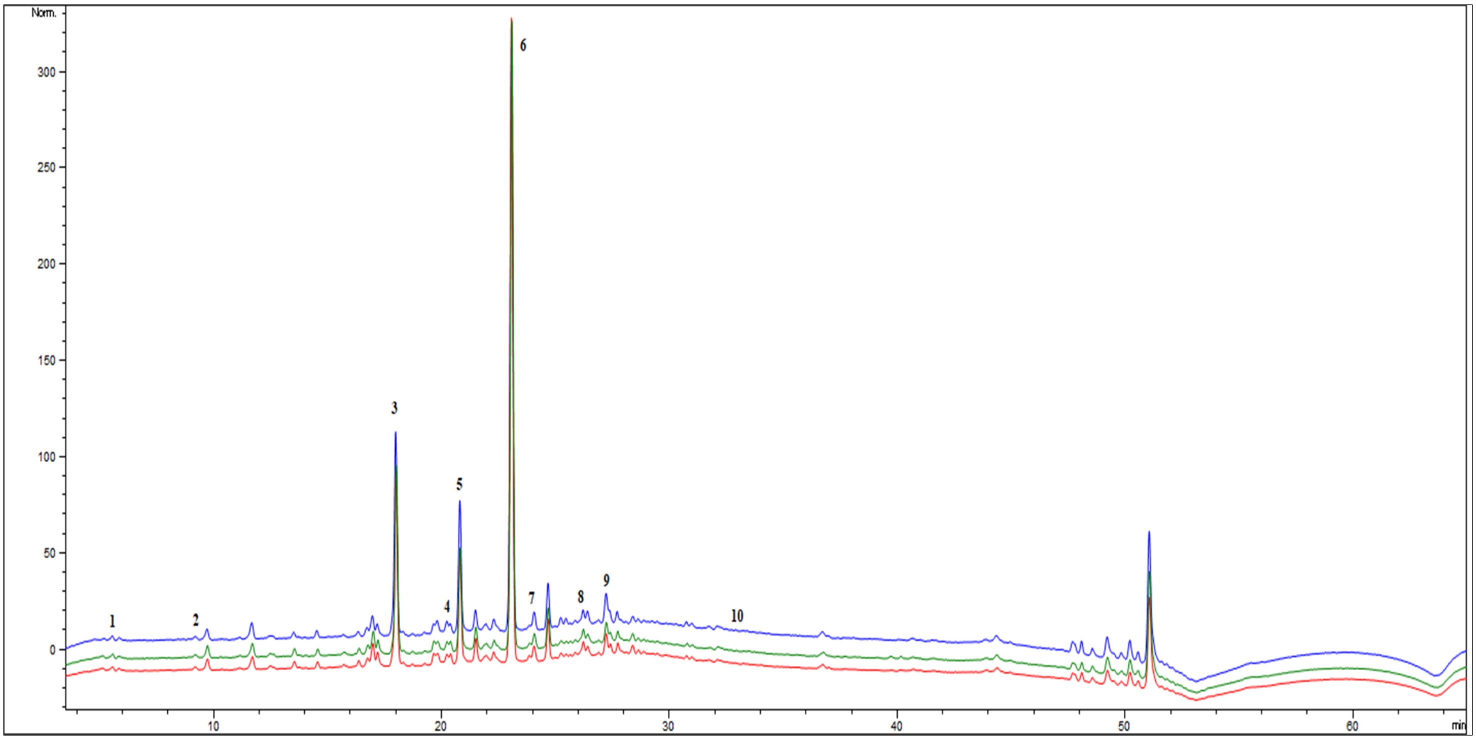

Figure S2. HPLC-DAD chromatograms of encapsulated extract liposome fractions with detection at 250 nm. Identified compounds: 1 - Hydroxytyrosol, 2 - Chlorogenic acid, 3 - Luteolin-7-O-glucoside, 4 - Quercitrin, 5 - Apigenin-7-O-glucoside, 6 - Oleuropein, 7 - Oleacein, 8 - Ligstroside, 9 - Quercetin, 10 - Oleuropein aglycone. AL - liposomes prepared using phospholipids from producer Avanti (red line), PG90 - liposomes prepared using granulated phospholipids from producer Lipoid (green line), and PH90 - liposomes prepared using hydrogenated phospholipids from producer Lipoid (blue line).

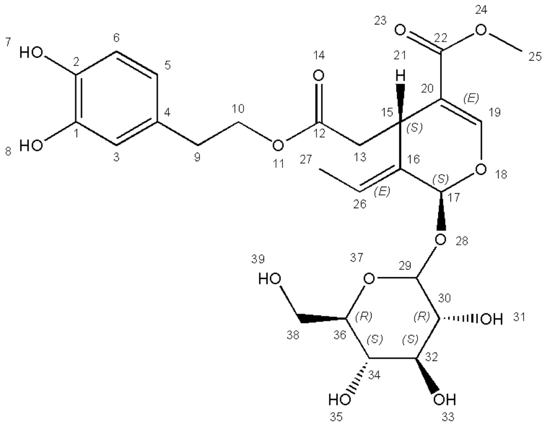

Oleuropein

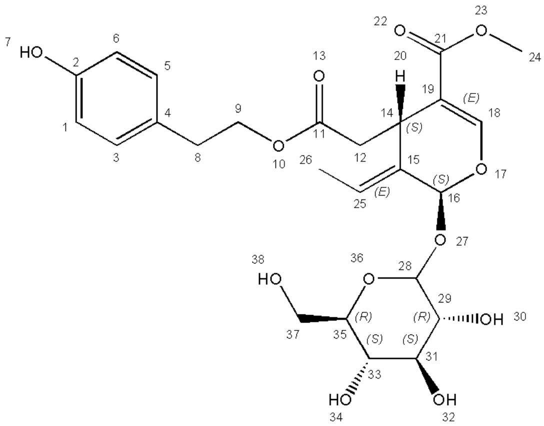

Ligstroisde

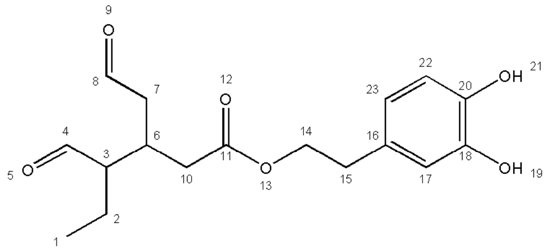

Oleacein

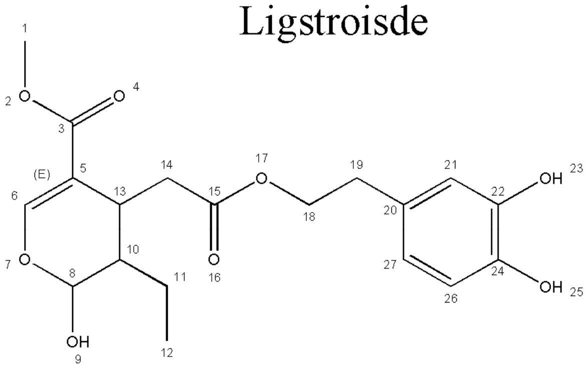

Oleuropein-aglycone

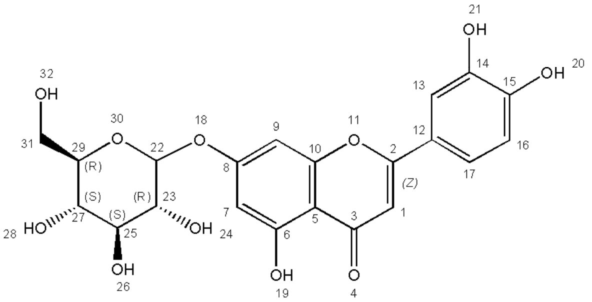

Luteolin-7-O-glucoside

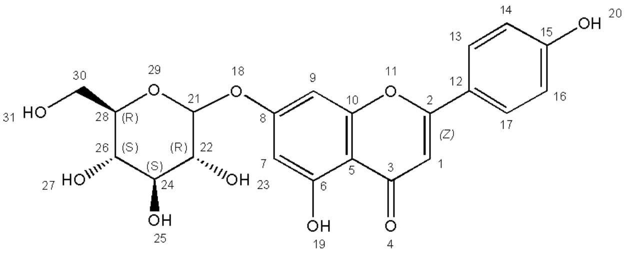

Apigenin-7-O-glucoside

Figure S3. Structural formulas of the main compounds in olive leaf extract: **Oleuropein** (Methyl (2S,3E,4S)-4-{2-[2-(3,4-dihydroxyphenyl)ethoxy]-2-oxoethyl}-3-ethylidene-2-{[(2S,3R,4S,5S,6R)-3,4,5-trihydroxy-6-(hydroxymethyl)oxan-2-yl]oxy}-2H-pyran-5-carboxylate); **Ligstroside** (Methyl (4S,5E,6S)-5-ethylidene-4-[2-[2-(4-hydroxyphenyl)ethoxy]-2-oxoethyl]-6-{[(2S,3R,4S,5S,6R)-3,4,5-trihydroxy-6-(hydroxymethyl)oxan-2-yl]oxy}-4H-pyran-3-carboxylate); **Oleacein** (2-(3,4-dihydroxyphenyl)ethyl (4Z)-4-formyl-3-(2-oxoethyl)hex-4-enoate); oleuropein-aglycone (Methyl (4S,5E,6R)-4-[2-[2-(3,4-dihydroxyphenyl)ethoxy]-2-oxoethyl]-5-ethylidene-6-hydroxy-4H-pyran-3-carboxylate); **Luteolin-7-O-glucoside** (2-(3,4-dihydroxyphenyl)-5-hydroxy-7-{[(2S,3R,4S,5S,6R)-3,4,5-trihydroxy-6-(hydroxymethyl)oxan-2-yl]oxy}-4H-chromen-4-one); **Apigenin-7-O-glucoside** (5-hydroxy-2-(4-hydroxyphenyl)-7-{[(2S,3R,4S,5S,6R)-3,4,5-trihydroxy-6-(hydroxymethyl)oxan-2-yl]oxy}-4H-chromen-4-one).

Table S2. Quantitative analysis of olive leaf extract and extract-loaded liposomes after the 60th day in the refrigerator.

Sample		Olive leaf extract	Extract-loaded liposomes		
			AL	PG90	PH90
Class of compounds	Compound	µg/mg dry extract			
Secoiridoids	Oleuropein	101.40±3.25[a]*	100.80±3.78[a]	37.82±1.47[c]	59.74±4.39[b]
	Oleacein	4.12±0.15[a]	3.31±0.12[b]	2.09±0.20[c]	3.06±0.56[b]
	Ligstroside	1.24±0.15[a]	0.98±0.09[b]	0.66±0.01[c]	1.06±0.22[ab]
	Oleuropein aglycone	0.53±0.08[a]	0.12±0.02[c]	0.25±0.05[b]	0.15±0.03[c]
Pentacyclic triterpenes	Oleanolic acid	40.10±2.10[a]	12.74±1.10[c]	20.08±1.6[b]	37.80±2.10[a]
	Maslinic acid	4.29±0.40[a]	0.15±0.03[d]	0.29±0.05[c]	2.90±0.20[b]
Flavonoids and flavonoid glycosides	Luteolin 7-O-glucoside	9.82±0.98[a]	8.38±0.34[b]	6.20±0.27[c]	10.67±0.86[a]
	Apigenin-7-O-glucoside	5.19±0.65[a]	3.08±0.55[b]	3.63±0.20[b]	5.81±0.11[a]
	Quercetin	0.61±0.20[c]	1.09±0.20[b]	0.94±0.09[b]	1.67±0.26[a]
	Quercitrin	0.31±0.05[a]	0.19±0.01[b]	0.13±0.03[c]	0.24±0.05[a]
Simple phenols	Hydroxytyrosol	1.86±0.32[b]	2.62±0.15[a]	0.52±0.05[d]	1.02±0.45[c]
	Chlorogenic acid	0.12±0.03[a]	0.03±0.01[b]	0.03±0.01[b]	0.03±0.01[b]

*The results are presented as an average value and standard deviation (Xm±S.D.) of three repeated measurements; AL - liposomes prepared using phospholipids from producer Avanti, PG90 - liposomes prepared using granulated phospholipids from producer Lipoid, and PH90 - liposomes prepared using hydrogenated phospholipids from producer Lipoid; GAE - gallic acid equivalents; d.e. - dry extract; the same letter in each row refers to the absence of statistically significant differences (for each compound separately) regarding the results of statistical analysis in one-way analysis of variance and Duncan's *post hoc* test at $p > 0.05$ ($n = 3$).

Table S3. Quantitative analysis of encapsulated extract fraction in liposomes and encapsulation efficiency after the 60th day in the refrigerator.

Sample		Encapsulated extract fraction in liposomes					
		AL		PG90		PH90	
Class of compounds	Compound	µg/mg d.e.	EE (%)	µg/mg d.e.	EE (%)	µg/mg d.e.	EE (%)
Secoiridoids	Oleuropein	70.77±3.54^{a}*	70.20±4.38^{b}	30.25±1.30^{c}	79.98±3.68^{a}	46.08±1.92^{b}	77.14±7.10^{ab}
	Oleacein	2.64±0.15^{b}	79.66±5.06^{a}	1.75±0.10^{c}	83.90±3.74^{a}	3.06±0.15^{a}	81.52±6.75^{a}
	Ligstroside	0.73±0.04^{a}	75.03±7.84^{a}	0.47±0.05^{b}	70.85±5.83^{a}	0.79±0.12^{a}	74.47±15.52^{a}
	Oleuropein aglycone	0.12±0.01^{b}	100±5.20^{a}	0.25±0.01^{a}	100±2.50^{a}	0.15±0.02^{b}	100±5.10^{a}
Pentacyclic triterpenes	Oleanolic acid	10.99±0.05^{c}	86.34±9.03^{b}	17.93±0.98^{b}	89.31±5.12^{b}	37.47±1.10^{a}	99.13±3.52^{a}
	Maslinic acid	0.09±0.01^{c}	61.20±14.14^{b}	0.24±0.01^{b}	82.56±9.10^{a}	2.90±0.20^{a}	100±11.30^{a}
Flavonoids and flavonoid glycosides	Luteolin 7-O-glucoside	7.39±0.41^{a}	88.00±6.06^{a}	5.62±0.15^{b}	89.79±4.35^{a}	7.75±0.29^{a}	73.00±5.41^{b}
	Apigenin-7-O-glucoside	2.84±0.21^{c}	92.13±12.80^{a}	3.37±0.14^{b}	92.75±6.40^{a}	4.77±0.10^{a}	82.15±4.62^{a}
	Quercetin	0.99±0.03^{b}	90.97±15.72^{a}	0.94±0.02^{b}	98.97±9.81^{a}	1.63±0.15^{a}	97.56±14.59^{a}
	Quercitrin	0.15±0.01^{b}	77.07±6.54^{a}	0.09±0.02^{c}	71.22±17.88^{a}	0.19±0.01^{a}	79.29±18.50^{a}
Simple phenols	Hydroxy tyrosol	1.29±0.07^{a}	49.36±3.89^{a}	0.25±0.02^{c}	49.31±8.97^{a}	0.66±0.02^{b}	64.78±12.39^{a}
	Chlorogenic acid	n.d.	0.00	n.d.	0.00	n.d.	0.00

*The results are presented as an average value (standard deviation) (Xm±S.D.) of three repeated measurements; EE - encapsulation efficiency, AL - liposomes prepared using phospholipids from producer Avanti, PG90 - liposomes prepared using granulated phospholipids from producer Lipoid, and PH90 - liposomes prepared using hydrogenated phospholipids from producer Lipoid; n.d. - not detected; d.e. - dry extract; the same letter in each row refers to the absence of statistically significant differences (for each variable, *i.e.*, concentration and EE, separately) regarding the results of statistical analysis in one-way analysis of variance and Duncan's post hoc test at $p > 0.05$ ($n = 3$).

A

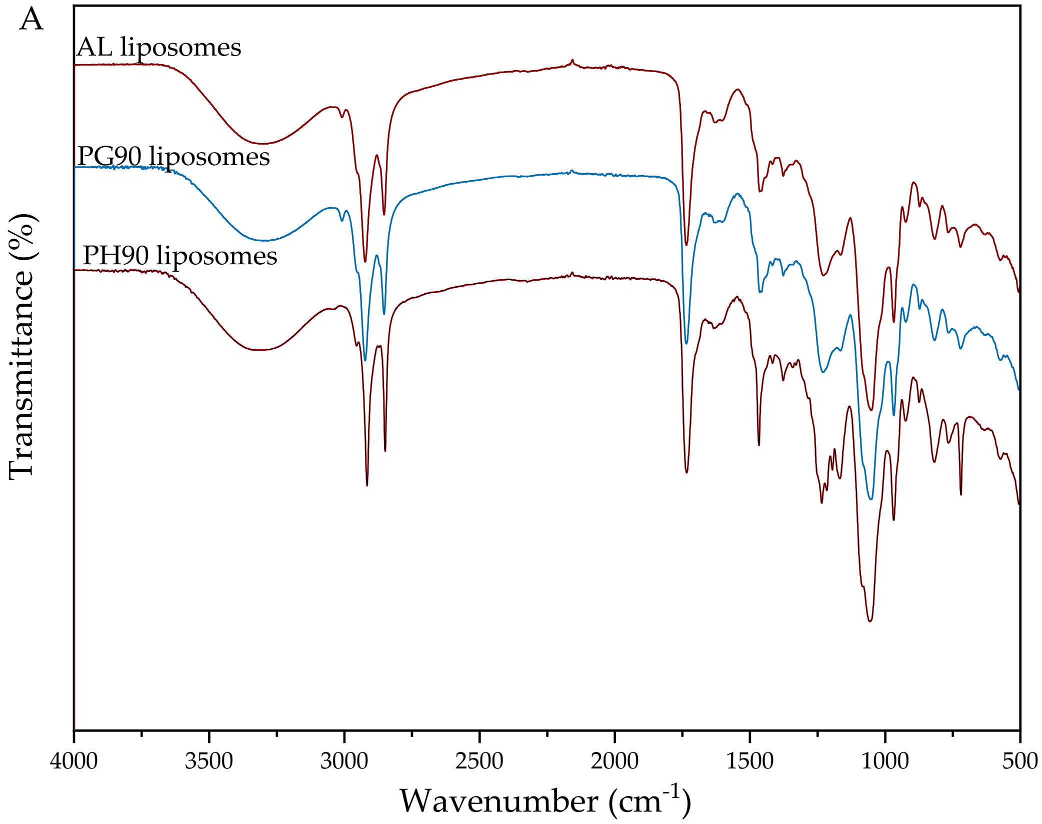

B

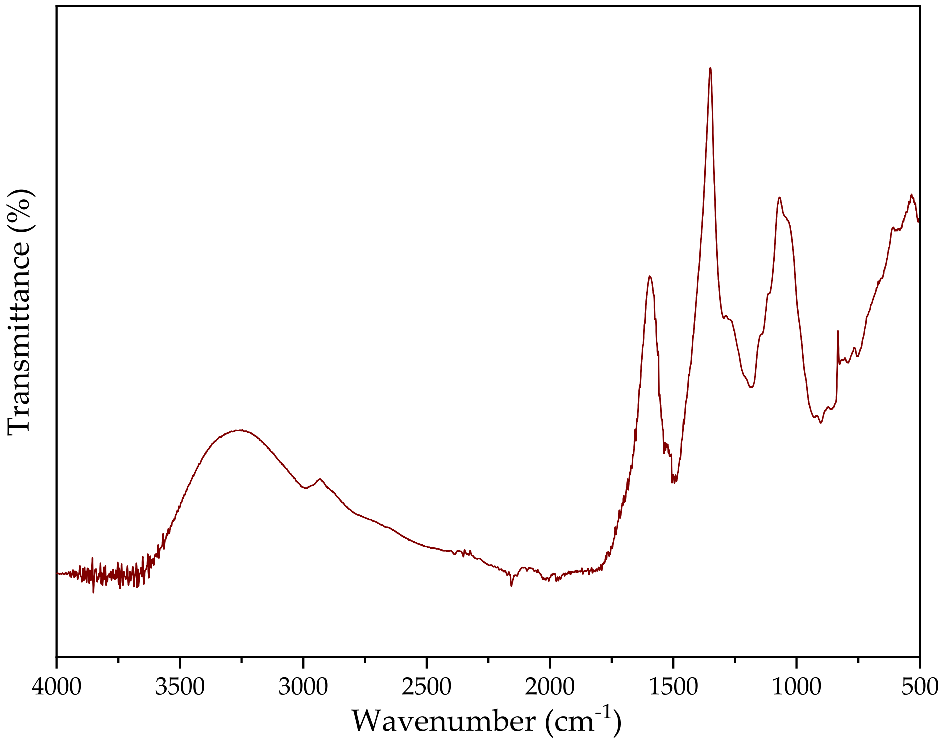

Figure S4. FT-IR spectra of (A) plain liposomes and (B) olive leaf extract; AL - liposomes prepared using phospholipids from producer Avanti, PG90 - liposomes prepared using granulated phospholipids from producer Lipoid, and PH90 - liposomes prepared using hydrogenated phospholipids from producer Lipoid.

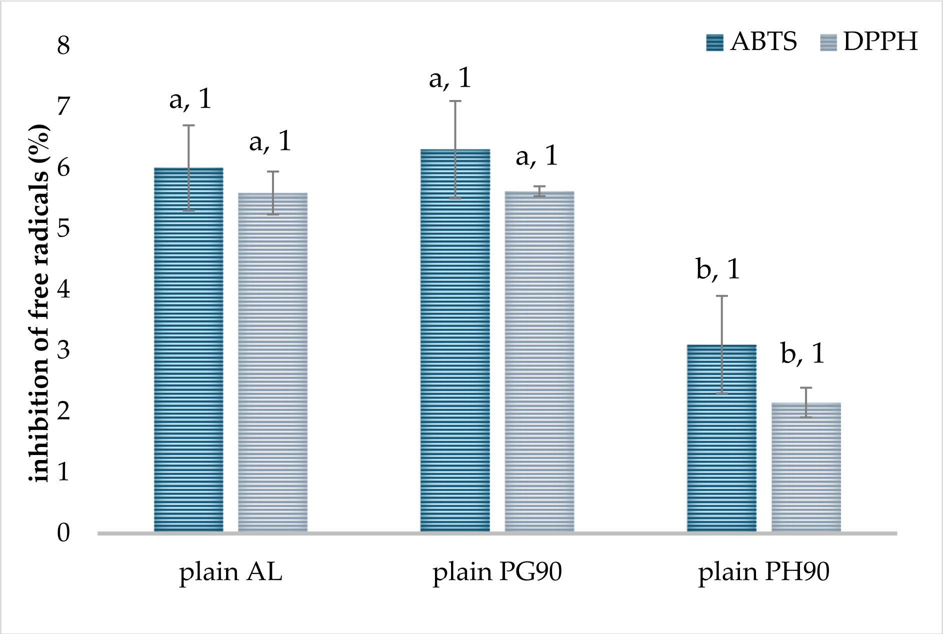

Figure S5. Antioxidant potential of plain liposomes; values with the same letter for each assay separately and the same number in each sample separately showed no statistically significant difference ($p > 0.05$; $n = 3$; analysis of variance, Duncan's *post hoc* test); AL - liposomes prepared using phospholipids from producer Avanti, PG90 - liposomes prepared using granulated phospholipids from producer Lipoid, and PH90 - liposomes prepared using hydrogenated phospholipids from producer Lipoid.

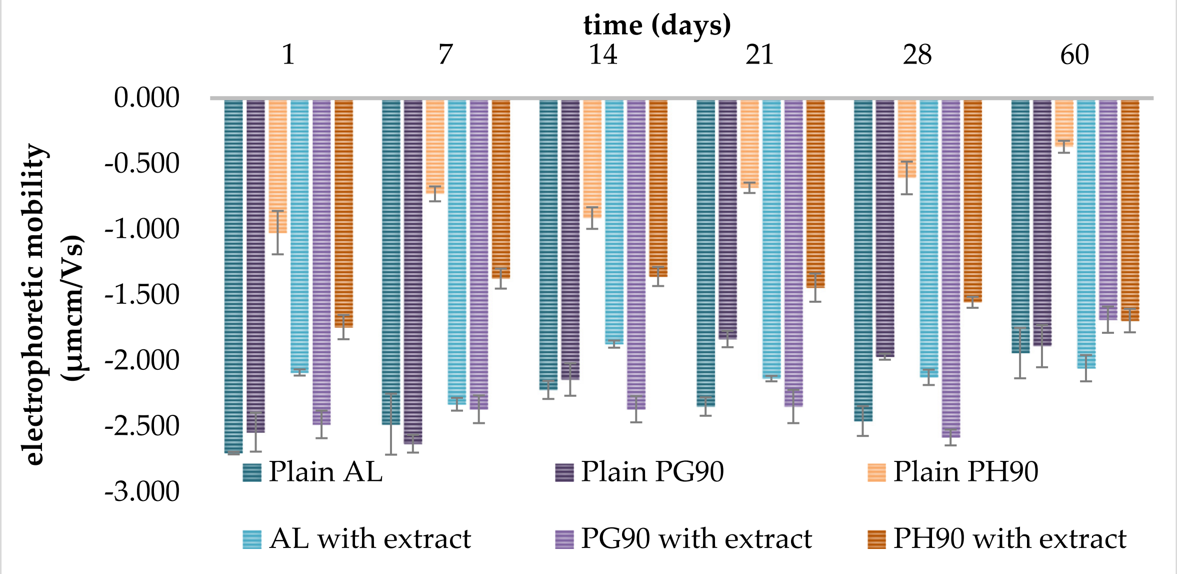

Figure S6. Electrophoretic mobility of plain and olive leaf extract-loaded liposomes over 60 days at 4°C; AL – liposomes prepared with phospholipids from Avanti; PG90 – liposomes with granulated phospholipids (Lipoid); PH90 – liposomes with hydrogenated phospholipids (Lipoid). Data represent mean±standard deviation of triplicate measurements.

Results obtained from the release studies of olive leaf extract and extract-loaded liposomes were analyzed to determine the diffusion coefficients (D) and diffusion resistances (R) derived from liposomes in simulated physiological conditions (phosphate buffer saline, pH 7.4, 37°C). The diffusion of polyphenols from liposomes to the receptor fluid through the membrane can be approximated using Fick's second law, shown in Equation (1):

$$\ln\left(\frac{C_d^0 - C_r^0}{C_d - C_r}\right) = D\,\beta\,t \quad (1)$$

where C_d and C_r are the concentrations of carob polyphenols detected in the donor and receptor compartments at time t; C_d^0 and C_r^0 are the concentrations of carob polyphenols at the beginning of the study; and D is the diffusion coefficient. The geometrical constant β value, typical for the Franz cell geometry, was 2.49×10^4 m^{-2}.

The diffusion coefficients of olive leaf polyphenols from liposome dispersion were calculated from the slope of the linear part of a curve defined by plotting $\ln\left(\frac{C_d^0 - C_r^0}{C_d - C_r}\right)$ *vs.* time.

The overall diffusion resistance, R, was calculated using Equation (2):

$$R = \frac{\delta}{D} \quad (2)$$

where δ is the membrane thickness.

Diffusion resistance represents the cumulative resistance of a semipermeable acetate cellulose membrane and the resistance of a liposomal bilayer. The contribution of the resistance, which is generated by the synthetic membrane, was determined from the diffusion of polyphenols from the pure olive leaf extract. Then, the liposome resistance was determined by subtracting the synthetic membrane resistance from the overall diffusion resistance.
